# A predictive multiphysics framework for optimizing focused ultrasound-assisted immune checkpoint blockade

**DOI:** 10.3389/fimmu.2026.1937764

**Published:** 2026-10-07

**Authors:** Marina Koutsi, Fotios Mpekris, Triantafyllos Stylianopoulos

**Affiliations:** 1 Cancer Biophysics Laboratory, Department of Mechanical and Manufacturing Engineering, University of Cyprus, Nicosia, Cyprus; 2 Cancer Genetics, Therapeutics and Ultrastructural Pathology Department, The Cyprus Institute of Neurology and Genetics, Nicosia, Cyprus

**Keywords:** cancer immunotherapy, computational model, drug delivery, tumor microenvironment, tumor perfusion

## Abstract

The efficacy of immune checkpoint blockers in solid tumors remains limited by the physical and biological barriers posed by the tumor microenvironment, which restrict antibody transport and suppress anti-tumor immune responses. Focused ultrasound has emerged as a promising strategy to transiently enhance tumor perfusion, thereby improving the intratumoral delivery of immunotherapeutic agents. Despite encouraging experimental findings, a quantitative understanding of the coupled transport and immune mechanisms underlying focused ultrasound-enhanced immunotherapy remains incomplete. Here, we developed a multiphysics computational model to investigate how focused ultrasound modulates the tumor microenvironment, enhances the delivery of immune checkpoint blockers and influences innate and adaptive immune responses in solid tumors. Model predictions were validated against published *in vivo* experimental data, demonstrating good agreement with tumor growth and immune response measurements. Parametric analysis identified both tumor microenvironment and therapy-related parameters as key determinants of treatment efficacy. Stromal normalization and increased vessel wall pore radius enhanced antibody delivery, immune cell accumulation and tumor control, while focused ultrasound further amplified these effects. Moreover, increasing the anti-PD-1/PD-1 binding affinity and antibody administration dose enhanced immune checkpoint inhibition and tumor suppression, with focused ultrasound broadening the range of antibody characteristics associated with favorable therapeutic outcomes. Overall, the proposed computational framework provides a predictive platform for investigating and optimizing focused ultrasound-assisted immune checkpoint blockade, offering mechanistic insight into how focused ultrasound-induced modulation of the tumor microenvironment enhances antibody transport, promotes anti-tumor immune responses and improves therapeutic response.

## 1 Introduction

Immunotherapy aims to boost the immune system to enhance cancer cell killing, in contrast to conventional chemotherapy, which directly targets cancer cells (1). Among immunotherapeutic strategies, immune checkpoint blockade (ICB) has emerged as one of the most successful approaches by inhibiting key immune regulatory pathways, including cytotoxic T-lymphocyte-associated protein 4 (CTLA-4), programmed cell death protein 1 (PD-1) and programmed death ligand 1 (PD-L1). By blocking these inhibitory pathways, ICB restores cytotoxic T cell function and promotes antitumor immune responses, leading to significant clinical benefits in several malignancies, particularly melanoma and non-small cell lung cancer (NSCLC) (2, 3). Despite these advances, fewer than 20% of cancer patients experience sustained clinical benefit (4), while immune-related adverse events remain a significant limitation (5). Therapeutic efficacy is particularly limited in solid tumors, where clinical studies have reported modest therapeutic responses to ICB (2). This limited efficacy is attributed to the highly immunosuppressive tumor microenvironment (TME) and the presence of the blood-brain barrier (BBB), which restricts the penetration of large therapeutic antibodies (6). Consequently, improving the intratumoral delivery and efficacy of ICB has become a major objective in cancer immunotherapy.

The abnormal characteristics of the TME pose a major barrier to the effective delivery and therapeutic efficacy of immune checkpoint blockers (ICBs) (7, 8). Solid tumors are highly heterogeneous tissues composed of malignant cells embedded within a complex TME, consisting of stromal cells, the vascular network and an abundant extracellular matrix (ECM) (9–13). During tumor progression, excessive deposition of ECM components, particularly collagen and hyaluronan, leads to desmoplastic remodeling, which increases tissue stiffness and generates solid stresses that compress intratumoral blood vessels (14–18). In addition, the often abnormal and highly permeable tumor vasculature elevates interstitial fluid pressure (IFP), reducing transvascular transport of therapeutic agents, while vascular compression impairs tumor perfusion and oxygen delivery, promoting hypoxia (12, 17, 19–23). Collectively, these abnormalities limit the intratumoral delivery and therapeutic efficacy of ICB while promoting tumor progression, immunosuppression and treatment resistance (7, 8, 24–26).

Focused ultrasound (FUS) has recently emerged as a promising non-invasive therapeutic modality for cancer treatment (27). By delivering acoustic energy with high spatial precision, FUS can induce both thermal and mechanical effects within targeted tissues (28, 29). Beyond its direct therapeutic effects, FUS has attracted considerable interest as a strategy for modulating the TME (27, 28). Through its mechanical effects, FUS can transiently increase tumor vascular permeability, enhance tissue perfusion and improve the intratumoral transport and accumulation of therapeutic agents, including ICBs (30–35). Furthermore, when combined with administration of microbubbles, FUS can safely and reversibly disrupt the BBB in brain tumors, thereby facilitating the delivery of large therapeutic molecules, such as ICBs (36, 37). Emerging evidence suggests that, beyond enhancing drug delivery, FUS may also modulate the immunosuppressive TME by promoting immune cell infiltration, enhancing antigen presentation and stimulating local inflammatory responses (38–40).

Preclinical and early-phase clinical studies further support the combination of FUS with ICB therapy. In glioblastomas, FUS combined with microbubbles transiently disrupted the BBB, enhancing tumor perfusion and anti-PD-1 delivery (37). In 4T1 murine breast tumors, short-pulsed FUS induced mechanical cavitation, reprogrammed the TME and improved the therapeutic efficacy of ICB (1, 27, 41). More recently, a combined preclinical and clinical study demonstrated that FUS-mediated BBB disruption improved the delivery and efficacy of doxorubicin and anti-PD-1 therapy in glioblastoma, accompanied by enhanced antitumor immune responses (42). These encouraging findings have led to several early-phase clinical trials investigating FUS in combination with ICBs, including pembrolizumab in metastatic breast cancer (43), the HILL (Ultrasound-Guided High-Intensity Focused Ultrasound Combined with PD-1 Blockade in Patients with Liver Metastases from Lung Cancer) study (44), and histotripsy combined with ipilimumab and nivolumab in metastatic or unresectable solid tumors (45).

Although these studies demonstrate the promising clinical potential of combining FUS with ICB, the mechanisms by which FUS-induced modulation of tumor perfusion enhances the intratumoral transport, distribution and therapeutic efficacy of ICBs remain incompletely understood. In this context, computational multiphysics modeling provides a powerful framework for elucidating these mechanisms and optimizing FUS-assisted immunotherapy. Numerous mathematical models have been developed to investigate FUS-mediated drug delivery by exploiting its thermal, mechanical, or combined effects to enhance intratumoral drug accumulation. However, these models have been primarily developed for chemotherapeutic applications, including temperature-sensitive liposomes (TSLs) (46–48) and other chemotherapeutic agents across different tumor types and treatment strategies (36, 49–56). In parallel, several mathematical models have been developed to investigate ICB and its interactions with cancer cells and the innate and adaptive immune systems (8, 57–59, 60–63). To the best of our knowledge, no integrated multiphysics mathematical framework has yet been reported to investigate the interplay between FUS-induced modulation of intratumoral transport and ICB.

To address this gap, we developed a three-dimensional multiphysics computational framework to investigate FUS-mediated potentiation of immune checkpoint blockade in solid tumors. Building on our previous work (46, 61, 62, 64–68), the proposed three-dimensional mechanistic tissue-level model integrates: (i) the effects of FUS-induced modulation of the TME through alterations in vascular permeability, vessel wall porosity and interstitial hydraulic conductivity and (ii) the transport and therapeutic action of ICB and (iii) its interactions with the innate and adaptive immune systems. The model further accounts for the structural and mechanical characteristics of the TME, including the solid stress-induced vessel compression, elevated interstitial fluid pressure and their effects on transvascular and interstitial transport. Model predictions are validated against published experimental data (27, 37) and parametric analyses are performed to identify the tumor and treatment characteristics governing therapeutic efficacy. In this context, the proposed framework provides a predictive platform for optimizing FUS-assisted ICB in solid tumors through the identification of favorable TME and treatment related conditions, rather than optimizing FUS acoustic exposure parameters.

## 2 Materials and methods

### 2.1 Modeled compartments and implementation

Tumor growth is described within a continuum mechanics framework based on the multiplicative decomposition of the deformation gradient tensor. The proposed deterministic model assumes quasi-static conditions and the equilibrium configuration of the tumor is obtained at each time step by solving the linear momentum balance equation (61, 62, 64–66, 69, 70). A detailed description of the mathematical formulation, governing equations, model assumptions and theoretical background is given in the **Supplementary Information (SI) Appendix**.

The model includes the complex interactions within the TME among multiple components that collectively contribute to the intricate process of tumor progression. Specifically, the model includes: (i) cancer cells (CCs) (62); (ii) innate and adaptive immune cell populations, including natural killer cells (NK), CD8^+^ T cells (C_d8_), CD4^+^ T cells (C_d4_), regulatory T cells (T_reg_) and M_1_ and M_2_ tumor-associated macrophages (TAMs); and (iii) physiological processes governing tumor perfusion, oxygen transport and the delivery of ICBs.

To investigate the effects of FUS, the model incorporates FUS-induced alterations in tumor transport properties. Specifically, FUS increases the effective vessel wall porosity, thereby enhancing vascular permeability (36). Furthermore, FUS exposure leads to a substantial increase in tumor hydraulic conductivity (36). These changes in vascular and interstitial transport properties are incorporated as mechanisms in the model. A schematic representation of these mechanisms is provided in **Figure 1**.

**Figure 1:**
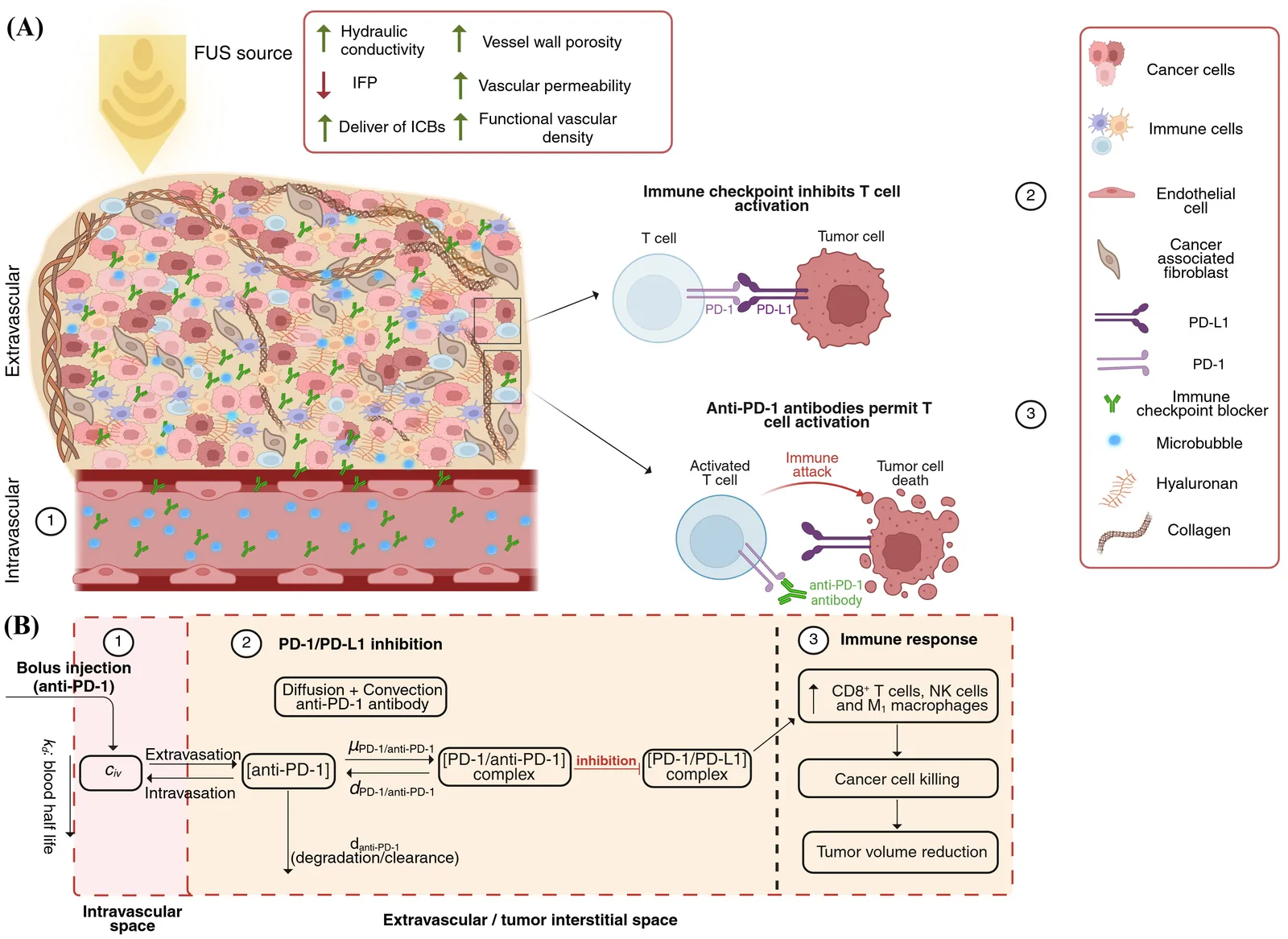
Schematic representation of the proposed multiphysics computational framework describing FUS-enhanced delivery of anti-PD-1 ICBs and the resulting anti-tumor immune response. **(A)** Biological mechanisms governing FUS-assisted ICB. Following intraperitoneal injection, anti-PD-1 antibodies circulate within the tumor vasculature. FUS in the presence of microbubbles transiently modifies the TME by increasing vessel wall porosity, vascular permeability, hydraulic conductivity and functional vascular density, while simultaneously reducing IFP. These transport-related changes enhance antibody extravasation and intratumoral accumulation within the tumor interstitium. (1) Enhanced transport of anti-PD-1 antibodies from the intravascular to the extravascular space through the tumor vessel wall. (2) In the absence of anti-PD-1 therapy, binding of PD-1 receptors expressed on T cells to PD-L1 ligands expressed on cancer cells forms the inhibitory PD-1/PD-L1 immune checkpoint complex, suppressing T cell activation and promoting tumor immune evasion. (3) Following anti-PD-1 administration, antibodies bind to PD-1 receptors, preventing PD-1/PD-L1 complex formation. Consequently, immune checkpoint inhibition is relieved, cytotoxic T cells recover their anti-tumor activity and immune-mediated killing of cancer cells is restored. **(B)** Representation of anti-PD-1 transport and immune checkpoint inhibition. The computational framework is organized into three interconnected stages. (1) Bolus injection: anti-PD-1 antibodies are administered intraperitoneally and transported across the tumor vessel wall according to Starling’s approximation, accounting for transvascular extravasation and intravasation. (2) PD-1/PD-L1 inhibition: anti-PD-1 antibodies undergo convection and diffusion within the tumor interstitium. Also, anti-PD-1 participates in reversible binding with PD-1 receptors to form the PD-1/anti-PD-1 complex. Antibody degradation and clearance are also incorporated. Formation of the PD-1/anti-PD-1 complex inhibits the formation of the inhibitory PD-1/PD-L1 complex. (3) Immune response: inhibition of PD-1/PD-L1 signaling restores the cytotoxic activity of CD8^+^ T cells, natural killer (NK) cells and M_1_ macrophages, thereby enhancing immune-mediated elimination of cancer cells. The resulting increase in cancer cell death leads to a reduction in total tumor volume (V_total_). Adapted from Koutsi et al. (46) under the CC BY 4.0 licence. Changes were made. Created with BioRender.com.

Within the proposed framework, FUS increases the functional vascular density, thereby enhancing tumor perfusion and promoting the intratumoral delivery of ICBs. The resulting increase in drug availability strengthens the immune-mediated elimination of cancer cells (14). Improved vascular function also enhances oxygen transport throughout the tumor, leading to elevated oxygen levels that support both tumor and immune cell activity. Furthermore, oxygen availability regulates the polarization of TAMs, promoting the transition from the immunosuppressive M_2_ phenotype toward the immunostimulatory M_1_ phenotype (61, 71, 72). However, rapid tumor growth generates solid stress that progressively compresses intratumoral blood vessels, thereby reducing tissue perfusion and promoting immune cell inactivation within the TME (22, 73). The model further accounts for the opposing roles of macrophage phenotypes in tumor progression, whereby M_1_ TAMs promote tumor cell elimination, whereas M_2_ TAMs suppress antitumor immune responses and maintain an immunosuppressive microenvironment (74, 75). A schematic overview of the mathematical model and interactions among its components is presented in **Supplementary Figure S1**, **SI Appendix**.

### 2.2 Description of mechanism of action of anti-PD-1 ICBs

#### 2.2.1 Interactions among PD-L1, PD-1 and anti-PD-1

PD-L1 concentration:

The concentration of free PD-L1 available for binding (57, 76, 77) is determined by the amount of PD-L1 expressed on the cell surface and the concentration of the PD-1/PD-L1 complex and is governed by the following equation:

(1) $$\left[\text{PD‐L}1]=\beta_{\text{CCs}}\cdot \text{CCs}+\beta_{\text{IM}}\cdot ({\text{M}}_{1}+{\text{M}}_{1})-[\text{PD‐}1/\text{PD‐L}1\right]$$

Equation 1 describes the correlation of PD-L1 expression with both immune cells (M_1_, *M*_2_ TAMs) and cancer cells (CCs), which are quantified by the coefficients *β*_*IM*_ for immune cells and *β*_CCs_ for cancer cells. Assuming that PD-1 and PD-L1 expression on each immune cell reaches a quasi-steady state, the total PD-L1 concentration is calculated as the sum of the contributions from immune cells and cancer cells. The free PD-L1 concentration, [PD-L1], is then obtained by subtracting the concentration of the PD-1/PD-L1 complex, [PD1/PD-L1], from the total PD-L1 concentration (here, square brackets [] represent the concentration of the receptor, ligand, antibody or complex while the slash/indicates the formation of a molecular complex). The PD-1/PD-L1 complex is formed through a one-to-one binding interaction between a PD-1 receptor and a PD-L1 ligand. Consequently, the remaining free PD-L1 is available for subsequent binding interactions. Although Equation 1 is formulated as an algebraic rather than a differential equation, the free PD-L1 concentration varies over time because both the cell populations and the concentration of the PD-1/PD-L1 complex are time-dependent.

PD-1 concentration:

The concentration of free PD-1 available for binding (57, 76, 77) is determined by the amount of PD-1 expressed on the cells and the concentration of the PD-1/PD-L1 complex and is described by the following equation:

(2) $$\left[\text{PD}‐1]={\text{α}}_{\text{IM}}({\text{C}}_{\text{d}8}+{\text{C}}_{\text{d}4}+\text{NK}+{\text{M}}_{1}+{\text{M}}_{1}+{\text{T}}_{\text{reg}})-[\text{PD‐}1/\text{PD‐L}1]-[\text{PD‐}1/\text{anti‐PD‐}1\right]$$

Equation 2 describes the concentration of PD-1, which includes contributions from multiple immune cell populations (C_d8_, C_d_*_4_*, *NK*, *M*_1_, *M*_2_ and *T*_*reg*_), quantified by the coefficient $a_{\text{IM}}$. The concentrations of the PD-1/PD-L1 and PD-1/anti-PD-1 complexes, denoted by [PD-1/PD-L1] and [PD-1/anti-PD-1], respectively, are subtracted from the total PD-1 concentration to account for receptor occupancy and determine the free PD-1 available for binding. These complexes are formed through one-to-one binding interactions between a PD-1 receptor and a PD-L1 ligand and between a PD-1 receptor and an anti-PD-1 antibody, respectively. Although Equation 2 is formulated as an algebraic rather than a differential equation, the free PD-1 concentration varies over time because both the immune cell populations and the concentrations of the bound complexes are time-dependent.

Dynamics of PD-1/PDL1 complex:

The rate of change in the concentration of the PD-1/PD-L1 complex is described by Equation 3:

(3) $$\frac{\text{d}[\text{PD‐}1/\text{PD‐L}1]}{\text{dt}}={\text{μ}}_{\text{PD‐}1/\text{PD‐L}1}[\text{PD‐}1][\text{PD‐L}1]-{\text{d}}_{\text{PD‐}1/\text{PD‐L}1}[\text{PD‐}1/\text{PD‐L}1]$$

The first term represents the formation of the PD-1/PD-L1 complex, whereas the second term describes its dissociation (57).

Dynamics of anti-PD-1/PD-1 complex:

The rate of change in the concentration of the anti-PD-1/PD-1 complex is described by Equation 4:

(4) $$\frac{\text{d}[\text{PD‐}1/\text{anti‐PD‐}1]}{\text{dt}}={\text{μ}}_{\text{PD‐}1/\text{anti‐PD‐}1}[\text{PD‐}1][\text{anti‐PD‐}1]-{\text{d}}_{\text{PD‐}1/\text{anti‐PD‐}1}[\text{PD‐}1/\text{anti‐PD‐}1]$$

where the first term is the formation of anti-PD-1/PD-1 complex and the second term is the dissociation of anti-PD-1/PD-1 complex (57).

Dynamics of anti-PD-1:

The spatiotemporal distribution of anti-PD-1 concentration is modeled using a convection-diffusion-reaction (CDR) equation, which describes the transport and kinetics of the ICBs and is expressed as:

(5) $$\frac{\partial [\text{anti‐PD‐}1]}{\partial \text{t}}+\nabla \cdot (‐{\text{D}}_{\text{anti‐PD‐}1}\nabla [\text{anti‐PD‐}1]+{\text{v}}^{f}[\text{anti‐PD‐}1])={\text{R}}_{\text{anti‐PD‐}1}={\text{γ}}_{\text{anti‐PD‐}1}-{\text{μ}}_{\text{PD‐}1/\text{anti‐PD‐}1}[\text{PD‐}1][\text{anti‐PD‐}1]\;+{\text{d}}_{\text{PD‐}1/\text{anti‐PD‐}1}\;[\text{PD‐}1/\text{anti‐PD‐}1]-{\text{d}}_{\text{anti‐PD‐}1}\;[\text{anti‐PD‐}1]+{\text{Q}}_{\text{sta}}$$

where D_anti-PD-1_ is the diffusion coefficient of the anti-PD-1 antibody within the tumor interstitial space. The reaction term, ${\text{R}}_{\text{anti‐PD‐}1}$, incorporates the principal biological processes governing antibody dynamics. Specifically, the first term, *γ*_*anti*−*PD*-1,_ represents the source of anti-PD-1 corresponding to its administration rate, whereas the second and third terms describe the formation and dissociation of the anti-PD-1/PD-1 complex, respectively. The fourth term accounts for the natural degradation and clearance of the antibody. Finally, the transport term, Q_sta_ describes the transvascular transport of the ICBs across the tumor vessel wall according to Starling’s approximation (57). The relative contributions of interstitial convection and diffusion to anti-PD-1 transport were further characterized using the Péclet number (Pe, Eq. S34). Near the tumor center and at therapy onset Pe ≈ 1.5×10^-3^, indicating that diffusion dominates over convection in the tumor interior under these conditions.

The term *Q_sta_* on the right hand side of Equation 5 represents the transport of the anti-PD-1 antibody across the tumor vessel wall and it is defined by Starling’s approximation as (21, 78, 79):

(6) $${\text{Q}}_{\text{sta}}={\text{P}}_{\text{er}}\cdot {\text{S}}_{V}\cdot ({\text{c}}_{\text{iv}}-[\text{anti‐PD‐}1])+{\text{L}}_{p}\cdot {\text{S}}_{\text{V}}\cdot (p_{\text{V}}-p_{\text{i}})\cdot (1-\sigma_{\text{f}}){\text{c}}_{\text{iv}}$$

where P_er_ is the vascular permeability, S_v_ the vascular density and *p*_i_ is the interstitial fluid pressure. Also, L_p_ denotes the hydraulic conductivity of the vessel wall, c_iv_ =exp (-(t-t_0_)/k_d_) represents the vascular concentration of the administered drug, which is indicative of a bolus injection, with t_0_ being the time of drug administration and k_d_ denoting the blood circulation decay, while σ_f_ is the reflection coefficient. The vascular conductivity L_p_ depends on the vessel wall pore radius, while the parameters P_er_ and σ_f_ vary with the ratio of the drug radius to the vessel wall pore radius (80). The right-hand side of Equation 6 accounts for two key mechanisms governing transvascular transport. The first term P_er_ S_v_ (c_iv_ –[anti-PD-1]) represents transvascular diffusion, driven by concentration gradients across the vessel wall, while the second term L_p_ S_v_ *p*_v_-*p*_i_) (1 - σ_f_) c_iv_ captures transvascular convection, driven by pressure differences between the vasculature and the interstitial space. Together, these mechanisms describe the extravasation of anti-PD-1 antibody from the blood vessels into the tumor tissue as well as from the tissue back into the circulation. A schematic representation of the aforementioned drug transport is given in **Figure 1B**.

The transvascular transport parameters, including the hydraulic conductivity of the vessel walls (Lp), vascular permeability (Per), and reflection coefficient (σ_f_), were calculated as functions of the vessel wall pore radius and the ratio of the drug particle size to the pore size, according to the theory of particle transport through cylindrical pores (80). The corresponding equations are provided in the **SI Appendix** (Eqs. S16-S23).

PD-1/PD-L1 complex distribution and fraction

The fractional distribution of the PD-1/PD-L1 complex formed between specific cells is given by *Φ*_x-y_ which refers to the formation of PD-1/PD-L1 complex from the binding of PD-1 receptor on x-cell to PD-L1 ligand on y-cell.

(7) $$\Phi_{\text{x-y}}=\frac{\alpha_{\text{x}}\text{x}}{\sum_{\text{x}}\alpha_{\text{x}}\text{x}}\frac{\beta_{\text{y}}\text{y}}{\sum_{\text{y}}\beta_{\text{y}}\text{y}}$$

Where x can be any of the following cell types which have PD-1 receptor on their extracellular membrane: C_d8_, C_d4_, *NK*, *M*_1_, *M*_2_ and T_reg_ and y can be any of the following cell types which have PD-L1 ligand on their extracellular membrane: CCs, *M*_1_ and *M*_2_ (57).

Equation 7 models the fraction of PD-1 expressed on cell type x that binds to PD-L1 expressed on cell type y, enabling the accurate representation of PD-1/PD-L1 complex formation among different cell populations within the TME. Coefficients *α*_*x*_ and *β*_*y*_ denote the expression levels of PD-1 and PD-L1 on each cell type, respectively and correspond to the coefficients introduced in Equations 1 and Equation 2 (*α*_*IM*_ for C_d8_, C_d4_, *NK*, *M*_1_, *M*_2_ and T_reg_, *β*_CCS_ for CCs and *β*_*IM*_ for *M*_1_ and *M*_2_). PD-1 is a critical immune checkpoint receptor involved in regulating the immune response, especially in the TME, and is expressed on several immune cell populations, including natural killer cells, CD8^+^ T cells, CD4^+^ T cells and *M*_1_ and *M*_2_ TAMs (81, 82), whereas PD-L1 is primarily expressed on cancer cells and macrophages (both *M*_1_ and *M*_2_) (82). The interaction between PD-1 and PD-L1 suppresses T-cell activity, thereby promoting tumor immune evasion (82). Consequently, blockade of this interaction by anti-PD-1 antibodies restores immune function and enhances the anti-tumor immune response (83). The parameter values used in this section for the incorporation of anti-PD-1 ICBs into the mathematical model, including the interactions among PD-L1, PD-1, and anti-PD-1, together with their units and corresponding references, are summarized in **Supplementary Table S1**, **SI Appendix**.

### 2.3 Components of the TME

The proposed mathematical model accounts for the dynamic interactions between cancer cells, immune cells and TAMs within the TME. A detailed description of these interactions is provided in the following subsections.

#### 2.3.1 Cancer cells

The equation governing the rate of change in cancer cells incorporates multiple biological processes, including tumor cell proliferation, represented by the term G·CCs and immune cell-mediated killing, represented by the remaining terms of the equation.

(8) $$\begin{array}{l} \frac{\partial \text{CCs}}{\partial \text{t}}+\nabla \cdot (‐{\text{D}}_{\text{CCs}}\nabla \text{CCs})={\text{R}}_{\text{CCs}}\\ =\text{G}\cdot \text{CCs}\\ -\underset{K_{r}}{\underset{︸}{{\text{K}}_{\text{rc}}(\begin{array}{l} (\frac{{\text{K}}_{\text{PD‐}1/\text{PD‐L}1}}{{\text{K}}_{\text{PD‐}1/\text{PD‐L}1}+\Phi_{\text{NK‐CCs}}[\text{PD‐}1/\text{PD‐L}1]})\cdot \text{NK+}\\ ({\text{n}}_{\text{ad}2\text{inn}}\frac{{\text{K}}_{\text{PD‐}1/\text{PD‐L}1}}{{\text{K}}_{\text{PD‐}1/\text{PD‐L}1}+\Phi_{{\text{C}}_{\text{d}8}\text{‐CCs}}[\text{PD‐}1/\text{PD‐L}1]})\cdot {\text{C}}_{\text{d}8}+\\ \;(\frac{{\text{K}}_{\text{PD‐}1/\text{PD‐L}1}}{{\text{K}}_{\text{PD‐}1/\text{PD‐L}1}+\Phi_{{\text{M}}_{1}\text{‐CCs}}[\text{PD‐}1/\text{PD‐L}1]})\;\cdot {\text{M}}_{1} \end{array})\cdot \text{CCs}}} \end{array}$$

where D_CCs_ denotes the diffusion coefficient of cancer cells, while R_CCs_ represents the reaction term governing cancer cell dynamics.

Cancer cell elimination is mediated by cytotoxic immune cells, including C_d8_ and NK cells through cytolysis and by M_1_ macrophages through phagocytosis. The inhibitory effects of PD-1/PD-L1 signaling are represented by the binding inhibition term


$$\left(\frac{{\text{K}}_{\text{PD‐}1/\text{PD‐L}1}}{{\text{K}}_{\text{PD‐}1/\text{PD‐L}1}+\Phi_{\text{x‐CCs}}[\text{PD‐}1\text{/PD}-\text{L}1]}\right)$$

which modulates the efficiency with which each immune cell population eliminates cancer cells.

As an example, the fraction of the PD-1/PD-L1 complex associated with C_d8_ is expressed as:


$$\begin{array}{l} \Phi_{\text{x‐CCs}}[\text{PD‐}1/\text{PD‐L}1],\text{ x}={\text{C}}_{\text{d}8}\\ \Phi_{{\text{C}}_{\text{d}8}\text{‐CCs}}[\text{PD‐}1/\text{PD‐L}1]=\\ \left(\frac{{\text{α}}_{\text{IM}}\cdot {\text{C}}_{\text{d}8}}{{\text{α}}_{\text{IM}}\cdot ({\text{C}}_{\text{d }8}+{\text{C}}_{\text{d}4}+\text{NK}+{\text{M}}_{\;1}+{\text{M}}_{\;1}+{\text{T}}_{\text{r e g}})}\cdot \frac{{\text{β}}_{\text{CCs}}\cdot \text{CCs}}{{\text{β}}_{CCs}\cdot \text{CCs}+{\text{β}}_{\text{IM}}\cdot ({\text{M}}_{\;1}+{\text{M}}_{\;1})})\;[\text{PD‐}1/\text{PD‐L}1\right] \end{array}$$

K_PD-1/PD-L1_ is the half-saturation constant of PD-1/PD-L1 complex and *Φ_x−CCs_*[PD-1/PD-L1] denotes the fraction of PD-1/PD-L1 complex formed between the immune cell type x and cancer cells (given by Equation 7), representing the distribution of binding across different interactions. C_d8_ have stronger killing potential than those of the innate system, thus their killing of cancer is assigned as n_add2inn_ times higher, where n_add2inn_ is a proportionality constant (57).

Cancer cell dynamics are governed by tumor proliferation and immune cell-mediated elimination (84). CD8^+^ T cells and natural killer cells eliminate cancer cells through cytolytic mechanisms (85). Additionally, phagocytosis of cancer cells by innate immune cells like M_1_ TAMs contribute to tumor suppression (85, 86). To account for tumor immune evasion, the inhibitory effects of the PD-1/PD-L1 signaling pathway are incorporated into the model, as this pathway suppresses immune cell-mediated killing of cancer cells (82).

The parameter G characterizes the proliferative capacity of cancer cells as a function of oxygen availability. The dependence of cancer cell proliferation on the local oxygen concentration is assumed to follow Michaelis–Menten kinetics, as described in Equation 9 (87, 88):

(9) $$\text{G=}\frac{{\text{k}}_{1}\;{\text{c}}_{\text{ox}}}{{\text{k}}_{1}+{\text{c}}_{\text{ox}}}$$

where k_1_ and k_2_ are parameters indicative of the growth rate, c_ox_ represents the concentration of oxygen.

#### 2.3.2 Modeling of the innate immune system

The innate immune response is represented by TAMs and NK cells. To capture the functional heterogeneity of macrophages within the TME, TAMs are classified into two phenotypes: the pro-inflammatory M_1_ macrophages and the anti-inflammatory M_2_ macrophages, each exerting distinct effects on tumor progression and immune regulation.

M_1_ macrophages

The temporal evolution of the M_1_ macrophage population is described by Equation 10, which incorporates three biological mechanisms: a gradual time-dependent activation, oxygen-dependent activation and natural degradation (61–63).

(10) $$\frac{\text{d }{\text{M}}_{\;1}}{\text{d t}}={\text{R}}_{\;{\text{M}}_{\;1}}=\underset{\begin{array}{l} \text{time-dependent}\\ \text{activation} \end{array}}{\underset{︸}{({\text{a}}_{\;{\text{M}}_{\;1}}\cdot \text{t })\cdot {\text{M}}_{\;1}}}+\underset{\begin{array}{l} \text{oxygen-dependent}\\ \text{activation} \end{array}}{\underset{︸}{{\text{k}}_{\;{\text{M}}_{\;1}\;{\text{O}}_{2}}\cdot \;{\text{oxrate}}_{\;{\text{M}}_{1}}\cdot {\text{M}}_{\;1}}}-\underset{\text{degradation}}{\underset{︸}{{\text{m}}_{{\text{ M}}_{\;1}}\cdot {\text{M}}_{\;1}}}$$

The time-dependent activation term represents the progressive recruitment and activation of M_1_ macrophages during tumor evolution, while the oxygen-dependent activation term accounts for the enhanced polarization of M_1_ macrophages under sufficiently oxygenated conditions. Specifically, oxygen availability (oxrate_M1_) is normalized by a reference oxygen concentration (c_ox,ref_ = 0.2 mol/m^3^), which represents the maximum oxygen concentration (i.e., the most oxygenated conditions) considered in the TME. This normalization yields a dimensionless oxygen ratio, enabling M_1_ activation to depend on relative rather than absolute oxygen concentrations (61, 89–91). This formulation reflects the pro-inflammatory phenotype of M_1_ macrophages, which is favored in well-oxygenated regions, whereas hypoxic conditions suppress their activation (62, 63). Finally, the degradation term represents the natural death and clearance of M_1_ macrophages, preventing unbounded population growth (62, 63).

M_2_ macrophages

The temporal evolution of the M_2_ macrophage population is described by Equation 11, which incorporates gradual time-dependent activation, hypoxia-dependent activation and natural degradation.

(11) $$\frac{d\;{\text{M}}_{2}}{d\text{ t}}={\text{R}}_{{\text{M}}_{2}}=\underset{\begin{array}{l} \text{time-dependent}\\ \text{ activation} \end{array}}{\underset{︸}{({\text{α}}_{{\text{ M}}_{2}}\cdot \text{t })\cdot {\text{M}}_{2}}}+\underset{\begin{array}{l} \text{hypoxia-dependent}\\ \text{ activation} \end{array}}{\underset{︸}{{\text{k}}_{{\text{M}}_{2}\text{ Hyp}}\cdot \;{\text{oxrate}}_{{\text{ M}}_{2}}\cdot {\text{M}}_{2}}}-\underset{\text{degradation}}{\underset{︸}{{\text{m}}_{M_{2}}\cdot {\text{M}}_{2}}}$$

The time-dependent activation term represents the gradual recruitment and activation of M_2_ macrophages during tumor progression, whereas the hypoxia-dependent activation term captures the preferential polarization of macrophages toward the M_2_ phenotype under low-oxygen conditions. Consequently, hypoxia enhances M_2_ activation, while improved oxygenation suppresses this response (61, 89–91). Finally, the degradation term represents the natural death, deactivation and clearance of M_2_ TAMs, preventing unbounded population growth.

NK cells

The temporal evolution of the NK cells population is described by Equation 12, which incorporates a time-dependent baseline activation term, vascular density-dependent recruitment through immune cell trafficking, natural degradation and immunosuppressive effects induced by Treg and M2 (61, 62).

(12) $$\begin{array}{l} \frac{d\text{ NK}}{d\text{ t}}={\text{R}}_{\text{NK}}=\underset{\begin{array}{l} \text{time-dependent }\\ \text{ activation} \end{array}}{\underset{︸}{{\text{s}}_{\text{N}{\text{K}}_{\text{base}}}\cdot \text{t}}}+\underset{\text{recruitment}}{\underset{︸}{{\text{s}}_{\text{NK}}\cdot {\text{T}}_{\text{f}}\cdot \left(1-\frac{\text{NK}}{\text{N}{\text{K}}_{\text{max}}}\right)}}-\underset{\begin{array}{l} \text{oxygen-dependent}\\ \text{ degradation} \end{array}}{\underset{︸}{{\text{f}}_{\text{NK}}\cdot \text{NK}}}\\ \;-\underset{\begin{array}{l} \text{inhibition of NK}\\ \text{ by }{\text{T}}_{\text{reg}}\; \end{array}}{\underset{︸}{{\text{l}}_{\text{Treg, NK}}\cdot {\text{T}}_{\text{reg}}\cdot \text{NK}}}-\underset{\begin{array}{l} \text{inhibition of NK}\\ \text{ by }{\text{M}}_{2} \end{array}}{\underset{︸}{l_{{\text{M}}_{2}\text{,NK}}{\text{M}}_{2}\cdot \text{NK}}} \end{array}$$

The baseline activation term represents the natural infiltration of NK cells into TME, whereas the vascular density-dependent recruitment term reflects the enhanced trafficking of NK cells following improvements in vascular functionality. NK recruitment is further regulated by a saturation term (1- NK/NK_max_) (63), preventing unlimited accumulation within the TME. The oxygen-dependent death term represents the reduced survival of NK cells under hypoxic conditions (71), while the inhibitory terms describe the suppression of NK cells mediated by Treg cells and M_2_ TAMs. A detailed description of the individual terms and the corresponding parameter values is provided in **Supplementary Table S2**, **SI Appendix**.

Trafficking of immune cells

Immunotherapy combined with FUS improves tumor perfusion and vessel porosity through modifications of the transport properties of the TME (36), leading to increased functional vascular density S_v_ (14, 16). These changes facilitate the infiltration of immune cells into the tumor. To account for this process, immune cell trafficking is modeled as a linear function of the functional vascular density, as described by Equation 13 (92):

(13) $$T_{f}=0.0273\cdot S_{v}-0.9138$$

where T_f_ is the immune cell trafficking function and S_v_ (cm^-1^) is the functional vascular density. The first term, 0.0273 ·S_v_ represents the enhancement of immune cell trafficking resulting from vascular density. Improvements in tissue perfusion, vessel porosity and functional vascular density facilitate the extravasation of immune cells from the vasculature, allowing them to migrate more efficiently into the tumor. The coefficient 0.0273 determines the sensitivity of immune cell trafficking to changes in vascular density. The constant term, -0.9138, shifts the trafficking function downward, introducing a threshold for effective immune cell infiltration. At low functional vascular density, the trafficking function remains close to zero, reflecting the limited ability of immune cells to penetrate poorly perfused tumors (92).

#### 2.3.3 Modeling of the adaptive immune system

In this mathematical model, the adaptive immune response is represented by CD8 ^+^ T cells, CD4^+^ T cells and regulatory T cells. These populations play complementary roles within the TME, with C_d8_ mediating cytotoxic anti-tumor activity, C_d4_ coordinating immune responses and Treg promoting an immunosuppressive TME by inhibiting anti-tumor immune responses.

Regulatory T cells (T_reg_)

The temporal evolution of Treg cells is described by Equation 14, which incorporates baseline activation, time-dependent recruitment and natural degradation.

(14) $$\frac{d\;{\text{T}}_{\text{reg}}}{d\text{ t}}={\text{R}}_{{\text{T}}_{\text{reg}}}=\underset{\text{ activation term}}{\underset{︸}{{\text{β}}_{{\text{T}}_{\text{reg}}}\cdot {\text{T}}_{\text{reg}}}}+\underset{\begin{array}{l} \text{time-dependent}\\ \text{ recruitment} \end{array}}{\underset{︸}{{\text{α}}_{{\text{T}}_{\text{reg}}}\cdot \text{t}\cdot {\text{T}}_{\text{reg}}}}-\underset{\text{degradation}}{\underset{︸}{{\text{m}}_{{\text{T}}_{\text{reg}}}\cdot {\text{T}}_{\text{reg}}}}$$

The baseline activation term maintains a constitutive T_reg_ population within the TME, whereas the time-dependent recruitment term represents the progressive accumulation of T_reg_ cells during tumor progression as the immunosuppressive environment becomes increasingly established. Finally, the degradation term accounts for the natural death and clearance of T_reg_. The T_reg_ death rate, m_Treg_, is modeled as a therapy-dependent parameter according to the expression (m_Treg_) (1+ if (t>t_treatment_,1,0)). Before treatment initiation (t≤t_treatment_), T_reg_ undergo their basal death rate. After therapy begins (t>t_treatment_), the conditional function doubles the death rate, representing the enhanced depletion of regulatory T cells induced by treatment (62, 93).

CD4^+^ T cells (C_d4_)

The temporal evolution of CD4+ T cells is described by Equation 15.

(15) $$\frac{d\;{\text{C}}_{d\;4}}{dt}={\text{R}}_{{\text{C}}_{\text{d}4}}=\underset{\text{recruitment}}{\underset{︸}{{\text{s}}_{{\text{C}}_{\text{d}4}}\cdot {\text{T}}_{\text{f}}\cdot (1-\frac{{\text{C}}_{\text{d}4}}{{\text{C}}_{\text{d}4_{\text{max}}}})\cdot {\text{C}}_{\text{d}4}}}-\underset{\text{degradation}}{\underset{︸}{{\text{m}}_{{\text{C}}_{\text{d}4}}\cdot {\text{C}}_{\text{d}4}}}$$

The recruitment term represents the basal infiltration of C_d4_ into TME, whereas the vascular density-dependent recruitment term reflects the enhanced trafficking of C_d4_ cells following improvements in vascular functionality. Following therapy initiation, CD4^+^ recruitment is further enhanced through a therapy-dependent activation term, (s_Cd4_) (1+ if (t>t_treatment_,1,0)) which accounts for the increased recruitment and activation of helper T cells induced by the combined effects of immunotherapy and FUS. C_d4_ recruitment is further regulated by a saturation term (1- C_d4_/C_d4max_) (63), preventing unlimited accumulation within the TME. Finally, the degradation term accounts for the natural death and clearance of C_d4_ (94).

CD8^+^ T cells (C_d8_)

The temporal evolution of CD8+ T cells is described by Equation 16.

(16) $$\begin{array}{l} \frac{d\;{\text{C}}_{d8}}{d\text{t}}={\text{R}}_{{\text{C}}_{d8}}=\underset{\text{recruitment}}{\underset{︸}{{\text{s}}_{{\text{C}}_{d8}}\cdot \;{\text{T}}_{\text{f}}\cdot \left(1-\frac{{\text{C}}_{d8}}{{\text{C}}_{d8max}}\right)}}-\underset{\text{degradation/death}}{\underset{︸}{{\text{f}}_{\text{C}}{\text{d}}_{8}\cdot {\text{C}}_{d8}}}\\ +\underset{\begin{array}{l} \text{stimulation of }{\text{C}}_{d8}\\ \text{ by }{\text{C}}_{d4} \end{array}}{\underset{︸}{{\text{r}}_{{\text{C}}_{d4}}\cdot \;{\text{C}}_{d4}\cdot {\text{C}}_{d8}}}+\underset{\begin{array}{l} \text{stimulation of }{\text{C}}_{d8}\\ \text{ by NK} \end{array}}{\underset{︸}{{\text{r}}_{\text{NK}}\cdot \text{ NK}\cdot {\text{C}}_{d8}}}-\underset{\begin{array}{l} \text{inhibition of }{\text{C}}_{d8}\\ \text{ by }{\text{T}}_{\text{reg}}\; \end{array}}{\underset{︸}{{\text{l}}_{{\text{T}}_{\text{reg}},{\text{C}}_{d8}}\cdot {\text{T}}_{\text{reg}}\cdot {\text{C}}_{d8}}}-\underset{\begin{array}{l} \text{inhibition of }{\text{C}}_{d8}\\ \text{ by }{\text{M}}_{2} \end{array}}{\underset{︸}{{\text{l}}_{{\text{M}}_{2},C_{d8}}\cdot {\text{M}}_{2}\cdot {\text{C}}_{d8}}} \end{array}$$

The temporal evolution of C_d8_ is governed by the recruitment term, defined as the product of the source term of C_d8_, the vascular density-dependent recruitment term reflects the enhanced trafficking of C_d8_ cells and the carrying-capacity term (1- C_d8_/C_d8max_), which prevents unlimited accumulation within the TME (63). In addition, the equation incorporates an oxygen-dependent death term, stimulatory interactions mediated by C_d4_ and NK cells and inhibitory interactions exerted by T_reg_ cells and M_2_ TAMs. A detailed description of the individual terms and parameter values is provided in **Supplementary Table S2**, **SI Appendix**.

### 2.4 Modeling the effects of FUS

The effects of FUS are incorporated into the mathematical model by accounting for its ability to transiently improve tumor perfusion through modifications of the transport properties of the TME. Experimental studies have demonstrated that FUS enhances vascular permeability by increasing the effective vessel wall porosity (*γ*) and improves interstitial fluid transport by increasing the hydraulic conductivity (*k_th_*) of tumor tissue (36). Accordingly, the hydraulic conductivity and effective vessel wall porosity are assumed to increase by factors of 5 and 1.5, respectively, following FUS exposure (36). These transport-related changes are considered to persist for 24 h after sonication (95), after which both parameters return to their baseline values. Mathematically, for a parameter par ∈ (γ, k_th_), we apply a step-like post-FUS modification, as described by Equation 17:

(17) $$par\;(t)=\{\begin{array}{c} par_{0},\;t<t_{FUS}\;\\ a_{p}\;par_{0},\;t_{FUS}\leq \;t<t_{FUS}+24\text{ h}\\ par_{0},\;t\geq t_{FUS}+24\text{ h} \end{array}$$

where, *par*_0_ denotes the baseline (pre-FUS) value (i.e., *k_th_*,_0_ for *k_th_* and *γ*_0_ for *γ*), *t_FUS_* is the sonication start time and *α_p_* = 5 and 1.5 are the multiplicative factors for hydraulic conductivity and vessel wall porosity, respectively.

The detailed description of the model and pertinent equations can be found in Eqs. (S1-S15), **SI Appendix** and the values of all model parameters, including their units and references, are summarized in **Supplementary Tables S1–S2**. The initial conditions of the variables at t=0 day are provided in **Supplementary Table S3**, **SI Appendix**.

### 2.5 Solution strategy

The computational framework was formulated in three spatial dimensions, with the tumor represented as a spherical domain embedded within a three-dimensional cubic domain of surrounding healthy tissue. The cubic host medium, which defined the spatial framework for tumor expansion, was configured to be two orders of magnitude larger than the tumor to minimize boundary-induced effects that could influence tumor evolution. Furthermore, geometric symmetry was assumed by modeling only one-eighth of the computational domain, thereby substantially reducing the computational cost while retaining its three-dimensional representation (96). The complete set of boundary conditions is provided in Eqs. (S24-S32) of the **SI Appendix**, while **Supplementary Figure S2** illustrates the computational domain and the corresponding boundary conditions. The model equations were solved using the commercial finite element software COMSOL Multiphysics 5.2a (COMSOL, Inc., Burlington, MA, USA). The computational mesh consisted of 6,015 free tetrahedral finite elements and 51,628 degrees of freedom. The finite element formulation employed quadratic Lagrange shape functions. Time integration was carried out via the Backward Differentiation Formula (BDF) scheme using the PARDISO solver and setting a time step of 0.25 d. The time-stepping approach was configured as Free, allowing the solver to automatically adapt the time increments to ensure numerical stability and convergence throughout the simulations. Mesh and time-step independence was assessed by repeating the simulations on a refined spatial discretization and with a reduced time step size. The methodology and corresponding results are provided in the Supporting Information and summarized in **Supplementary Table S5**, **SI Appendix**. In addition, for the convection-diffusion-reaction equation (Equation 5) governing anti-PD-1 transport, COMSOL’s consistent stabilization was employed, with both streamline diffusion and crosswind diffusion enabled to enhance numerical stability in regions where convective transport may become locally important.

## 3 Results

### 3.1 Validation of the computational model

The model’s predictive performance was evaluated by comparing model predictions with measurements from published experimental data of *in vivo* studies (27, 37). To enable a direct comparison between model predictions and experimental observations, all model parameters were assigned baseline values according to the literature (**Supplementary Tables S1–S2**, **SI Appendix**). The only parameter adjusted to align the model predictions with experimental results was the parameter denoted as k_1_, which quantifies the relationship between cancer cell proliferation and oxygen concentration (Equation 9). The value of k_1_ was determined by fitting the tumor growth data of the untreated (control) group and was subsequently held constant across all treatments within the same study. Specifically, k_1_ was estimated as 0.33 day^-1^ for C6 glioma cells (37) and 0.385 day^-1^ for 4T1 breast cancer cells (27) (**Supplementary Table S4**; **SI Appendix**). An optimization algorithm (92, 97) was employed to minimize the error between experimental data and model predictions. Since each tumor cell line exhibits distinct growth dynamics, calibrating k_1_ for each tumor type ensures that the model accurately captures the biological variability in tumor proliferation rates.

An *in vivo* study using a C6 glioma model demonstrated that FUS combined with microbubbles transiently opened the BBB, enhancing tumor perfusion and improving the intratumoral delivery of anti-PD-1 ICBs. As a result, the combined treatment significantly enhanced the therapeutic efficacy of ICB and promoted a more favorable anti-tumor immune microenvironment by alleviating glioma-associated immunosuppression (37). To validate the proposed mathematical model, simulations were performed under the same experimental protocol and results are presented in **Figure 2A**. Briefly, mice bearing C6 glioma tumors were randomly assigned to three treatment groups: Control, ICBs and FUS+ICBs. Treatment was initiated once tumors reached a volume of approximately 40 mm³ (day 11). The Control group received sham FUS without acoustic energy, whereas the ICBs group received intraperitoneal anti-PD-1 administration (2 mg/kg) on experimental days 11, 13, 15 and 17. The FUS+ICBs group received the same anti-PD-1 regimen combined with FUS treatment (4 W, 200 s) on the corresponding treatment days. Tumors were isolated on day 19 for analysis.

**Figure 2:**
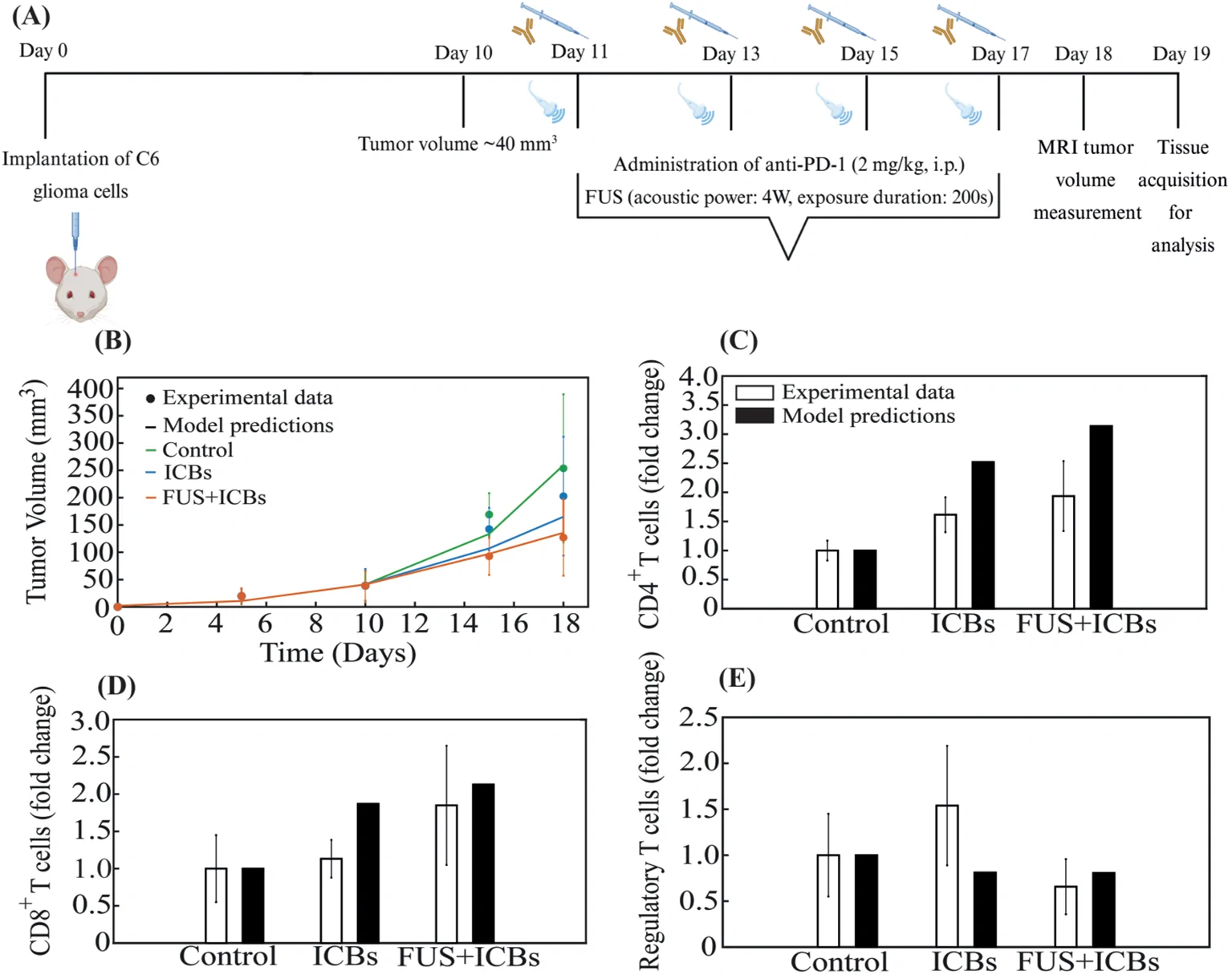
Comparison between model predictions and experimental data for tumor growth and adaptive immune cell populations in C6 glioma tumors (37). **(A)** Experimental treatment protocol applied to C6 glioma cells and replicated in the model simulations. Created with BioRender.com. **(B)** Tumor growth of C6 glioma cells (dots) compared with model predictions (solid lines) for each treatment group. Model performance was evaluated using the coefficient of determination (R²) and RMSE: Control: R² = 0.97, RMSE = 16.55 mm^3^; ICBs: R² = 0.91, RMSE = 23.43 mm^3^; FUS+ICBs: R² = 0.98, RMSE = 5.89 mm^3^. **(C–E)** Fold changes in CD4^+^ T cells, CD8^+^ T cells and regulatory T cells, respectively, for the control, ICBs and FUS+ICBs groups. Immune cell populations are presented as fold changes relative to the control group. White bars represent experimental data and black bars represent model predictions. Error bars denote standard error of the mean (SE) for experimental data (n = 7). For the simulations, immune cell populations were evaluated on day 19, corresponding to the day of experimental tissue harvesting, for all treatment groups.

Overall, the proposed model reproduced the experimental observations with good accuracy, demonstrating strong agreement with both tumor growth and adaptive immune cell responses (**Figure 2**). As shown in **Figure 2B**, the predicted tumor growth curves closely matched the experimental measurements for all treatment groups, with the combined FUS+ICBs therapy producing the greatest inhibition of tumor progression. The corresponding coefficients of determination (R²) were 0.97, 0.91 and 0.98 for the Control, ICBs and FUS+ICBs groups, respectively, further confirming the predictive capability of the model. The corresponding RMSE values were 16.55, 23.43 and 5.89 mm³, respectively. The higher RMSE and the lower R^2^ for the ICBs group were mainly associated with an underprediction of tumor volume at days 15 and 18. It should be noted that the tumor proliferation parameter was calibrated only against the Control tumor growth data and was subsequently kept fixed across all other treatment groups, which should contribute to the accuracy of the fitting. To enable direct comparison between the computational and experimental results, adaptive immune cell populations were expressed as fold changes relative to the control group. As shown in **Figure 2C**, both the experimental measurements and model predictions demonstrated increased CD4^+^ T cell accumulation following treatment, with the largest increase observed in the FUS+ICBs group. However, the model overpredicted the magnitude of the CD4^+^ T cell increase in both treatment groups. A similar trend was observed for CD8^+^ T cells (**Figure 2D**), where the combined therapy produced the strongest cytotoxic T-cell response, indicating enhanced anti-tumor immunity. The dynamics of regulatory T cells are presented in **Figure 2E**. Both the experimental data and the model predicted an overall reduction in T_reg_ population following the combined FUS+ICBs treatment, consistent with inhibition of the immunosuppressive tumor microenvironment. The overall agreement between the experimental and predicted immune cell fold changes was quantified using RMSE, yielding a value of 0.76. Control values were excluded from this calculation because they represent the reference values used for data normalization. Although the experimental data showed a slight increase in T_reg_ in the ICBs group, the overall trend remained consistent with the model predictions. This transient increase has also been reported experimentally and has been associated with an unfavorable prognosis, as T_reg_ suppress the activation and function of effector immune cells through multiple mechanisms, thereby promoting tumor immune evasion and reducing anti-tumor immunity (37, 98, 99).

Additionally, data from another *in vivo* study using 4T1 murine breast tumors was employed to further validate the proposed mathematical model (27). This experimental study demonstrated that combining FUS with anti-PD-1 ICB significantly enhanced the therapeutic response in triple-negative breast cancer. FUS transiently increased vascular permeability, thereby facilitating intratumoral drug delivery and improving immune modulation. These findings highlight the potential of combining FUS with immunotherapy as a strategy to enhance treatment efficacy in breast cancer and other solid tumors (27). The mathematical model was formulated to reproduce the experimental protocol shown in **Figure 3A**. Briefly, mice were randomly assigned to three treatment groups: Control, ICBs, and FUS+ICBs. Treatment was initiated on experimental day 10. The ICBs group received anti-PD-1 antibodies (200 μg) on days 10, 13 and 16, whereas the FUS+ICBs group received the same anti-PD-1 regimen combined with FUS treatment (2.8 kW/cm², 10 s) on days 10 and 13. Tumors were isolated on day 19 for analysis, while the experiment was terminated once tumor volumes reached approximately 1000 mm³.

**Figure 3:**
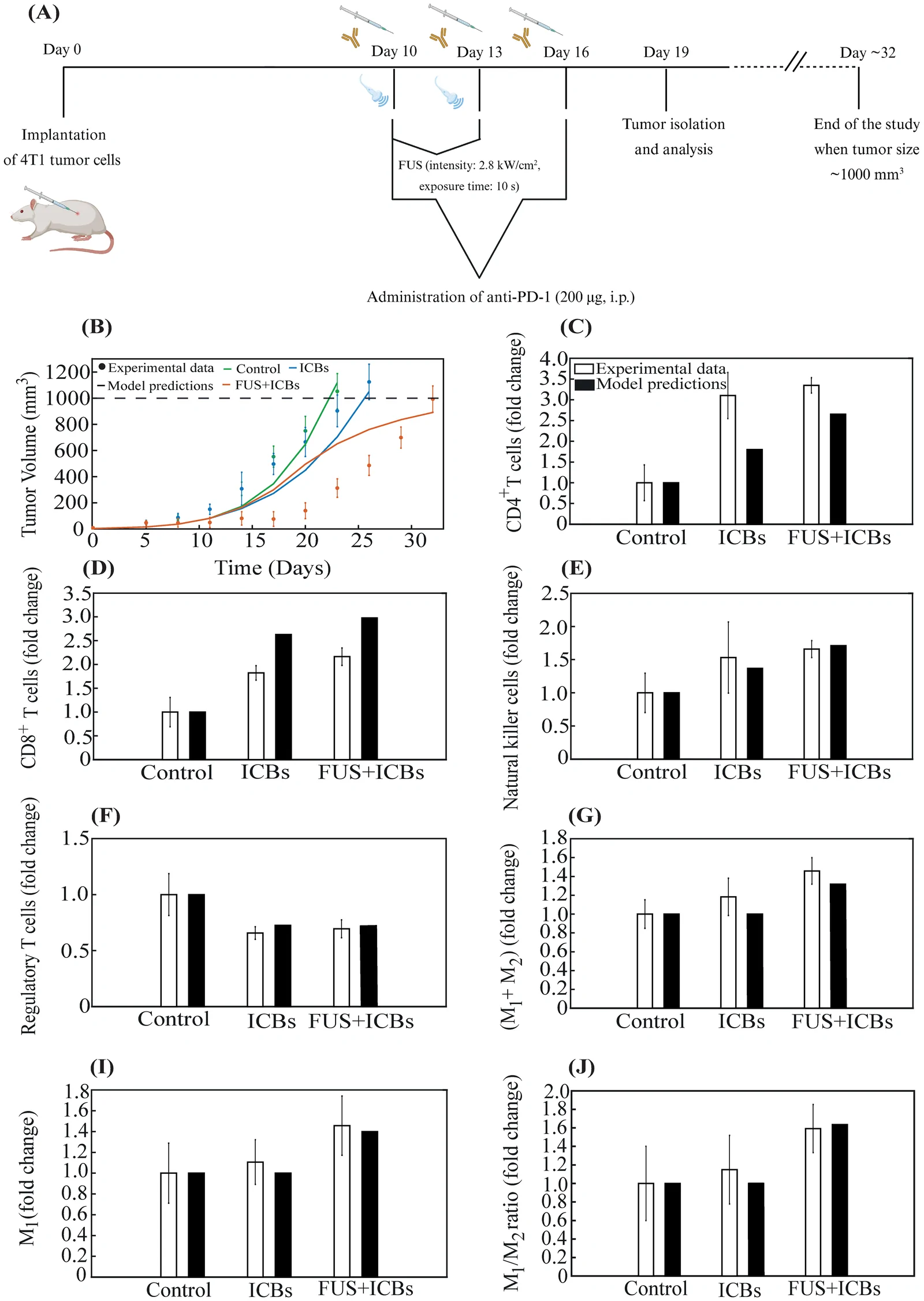
Comparison between model predictions and experimental data for tumor growth and innate and adaptive immune cell populations in 4T1 breast tumors (27). **(A)** Experimental treatment protocol applied to 4T1 breast tumors and replicated in the model simulations. Created with BioRender.com. **(B)** Tumor growth of 4T1 cells (dots) compared with model predictions (solid lines) for each treatment group. Model performance was evaluated using the coefficient of determination (R²) and RMSE: Control: R² = 0.92, RMSE = 102.74 mm^3^; ICBs: R² = 0.87, RMSE = 138.92 mm^3^; FUS+ICBs: R² = 0.70, RMSE = 177.94 mm^3^. **(C–J)** Fold changes in CD4^+^ T cells, CD8^+^ T cells, natural killer cells, regulatory T cells, total TAMs (M_1_+M_2_), M_1_ TAMs and the M_1_/M_2_ ratio respectively, for the control, ICBs and FUS+ICBs groups. Immune cell populations are presented as fold changes relative to the control group. White bars represent experimental data and black bars represent model predictions. Error bars denote standard error of the mean (SE) for experimental data (n = 8). For the simulations, immune cell populations were evaluated on day 19, corresponding to the day of experimental tissue harvesting, for all treatment groups.

As shown in **Figure 3B**, the simulated tumor growth curves closely reproduced the experimental measurements for all treatment groups. The corresponding coefficients of determination (R²) were 0.92, 0.87 and 0.70, while the RMSE values were 102.74, 138.92 and 177.94 mm^3^ for the Control, ICBs and FUS+ICBs groups, respectively, further confirmed the predictive capability of the model. The lower R^2^ value for the FUS+ICBs group resulted primarily from an overestimation of tumor growth between days 14 and 29. During this period, the experimental tumors exhibited a slower and more gradual increase in volume, whereas the model predicted a steeper growth. This difference should be related to the current simulation of FUS, which primarily accounts for FUS-induced changes in tumor transport properties and the resulting enhancement of anti-PD-1 delivery. Additional or prolonged FUS-mediated biological effects on the TME (27, 37, 42, 100), including modulation of local immune responses through changes in immune cell recruitment and activation, as well as alterations in inflammatory and cytokine signaling within the TME (27), could contribute to the sustained tumor suppression observed experimentally but are not explicitly incorporated into the present framework. The absence of these additional biological effects may therefore contribute to the steeper tumor growth predicted by the model compared with the experimental data. Nevertheless, the model captured the early response, approached the experimental tumor volume at the final time point and correctly predicted the greatest tumor inhibition for the combined treatment. To facilitate direct comparison between the computational and experimental results, immune cell populations were expressed as fold changes relative to the control group. As shown in **Figure 3C** and **Figure 3D**, both the experimental measurements and model predictions showed increased CD4^+^ and CD8^+^ T cell populations following treatment, with the largest increase observed in the FUS+ICBs group, indicating enhanced adaptive anti-tumor immunity. For CD4^+^ T cells, however, the model underpredicted the magnitude of the experimentally observed increase, particularly for the ICBs group. Similarly, both the experimental data and the simulations showed increased NK cell populations following ICBs and FUS+ICBs treatment (**Figure 3E**), suggesting enhanced innate immune activation. The dynamics of regulatory T cells are presented in **Figure 3F**. Both the experimental observations and the model predictions showed reduced T_reg_ populations following ICBs and FUS+ICBs treatment, indicating a partial reversal of the immunosuppressive TME. The macrophage-related results are shown in **Figures 3G–J**. Both the computational predictions and the experimental data demonstrated increases in the total TAMs population (M_1_+M_2_), M_1_ macrophages and the M_1_/M_2_ ratio following treatment, with the largest changes observed in the combined FUS+ICBs group. Conversely, the M_2_ macrophage population decreased after treatment, reflecting macrophage polarization toward the pro-inflammatory M_1_ phenotype and a shift toward a more immunostimulatory TME. The overall RMSE between the experimental and predicted immune cell fold changes was 0.52, excluding the Control values, which were used for data normalization.

Overall, the mathematical model reproduced the main experimentally observed trends in tumor growth and immune cell responses across all treatment groups of both studies, while the quantitative discrepancies described above indicate limitations in capturing the magnitude of some treatment-induced responses. In particular, the combined FUS+ICBs therapy resulted in the greatest tumor suppression together with enhanced infiltration of anti-tumor immune cells and reduced immunosuppressive cell populations, demonstrating the enhanced therapeutic effects of FUS+ICBs.

### 3.2 Parametric analysis

A parametric analysis was performed to investigate the effect of key model parameters on tumor progression and treatment response. A comprehensive global sensitivity analysis of the principal parameters governing tumor growth modeling was previously conducted (101, 102). However, that study did not account for the effects of FUS or immune ICBs. Therefore, in the present work, a parametric analysis was performed using the coupled FUS-enhanced perfusion and ICBs delivery model to evaluate the influence of parameters specifically associated with TME, including the tissue elastic modulus (E) and the vessel wall pore radius (r_o_), as well as treatment-related parameters, namely the binding affinity between PD-1 and anti-PD-1 (*μ*_PD-1/anti-PD-1_) and the anti-PD-1 administration rate (*γ*_anti-PD-1_).

#### 3.2.1 Impact of TME parameters on treatment outcomes

To investigate whether normalization of the tumor stroma can further improve the therapeutic efficacy of ICB, stromal normalization was incorporated into the mathematical model based on the mechanotherapeutic framework (14, 103). Specifically, stromal normalization reduces solid stress levels within the tumor, by reducing tissue stiffness, which as a result decompresses tumor blood vessels and improves vascular perfusion. Experimental studies have shown that approximately three days after administration of stromal normalization agents, tumor stiffness is reduced by nearly 50%. Concurrently, tumor perfusion increases, while the hydraulic conductivity of the tumor interstitial space exhibits a linear increase of two orders of magnitude compared with its initial value in the absence of stromal normalization, thereby facilitating drug transport and immune cell trafficking. These experimentally observed changes were incorporated into the model by progressively reducing the tumor elastic and bulk moduli to represent the decrease in tissue stiffness, while simultaneously increasing the hydraulic conductivity (14, 16, 104–106).

In the proposed parametric analysis, stromal normalization was initiated three days before the first ICBs administration (defined as day 0 in the days post-treatment x axis of **Figure 4**) and was administered daily throughout the treatment period (14, 103). ICBs (or FUS+ICBs) were subsequently administered on treatment days 3, 6 and 9. This scheduling allowed the stroma to be remodeled before immunotherapy delivery, thereby improving vascular functionality at the time of drug administration (104). As shown in **Figure 4A**, stromal normalization enhances the intratumoral concentration of ICBs in both treatment strategies. This improvement is more pronounced when stromal normalization is combined with FUS, since FUS transiently increases vessel wall porosity and vascular permeability, whereas stromal normalization softens tumors and improves perfusion and interstitial resistance to transport. Consequently, the combination of these complementary mechanisms results in the greatest accumulation of therapeutic antibodies within the tumor. The increased intratumoral drug availability is accompanied by a more pronounced inhibition of tumor growth (**Figure 4B**). Among all treatment strategies, the combined FUS+ICBs+stromal normalization protocol produces the greatest reduction in tumor volume, demonstrating the enhanced benefit of simultaneously enhancing vascular transport, drug penetration and immune activation. The effects of stromal normalization are also reflected in the predicted immune cell dynamics. As shown in **Figures 4C–E**, stromal normalization promotes a more immunostimulatory TME by increasing the M_1_/M_2_ macrophage ratio, natural killer cells and CD8^+^ cytotoxic T cells. These increases are particularly evident for the FUS+ICBs+stromal normalization treatment, indicating that improved vascular functionality facilitates immune cell recruitment into the tumor while simultaneously enhancing the effectiveness of ICB. As a result, a larger fraction of activated immune cells is able to bind anti-PD-1 antibodies instead of interacting with PD-L1 expressed by tumor cells, thereby restoring their cytotoxic activity against cancer cells.

**Figure 4:**
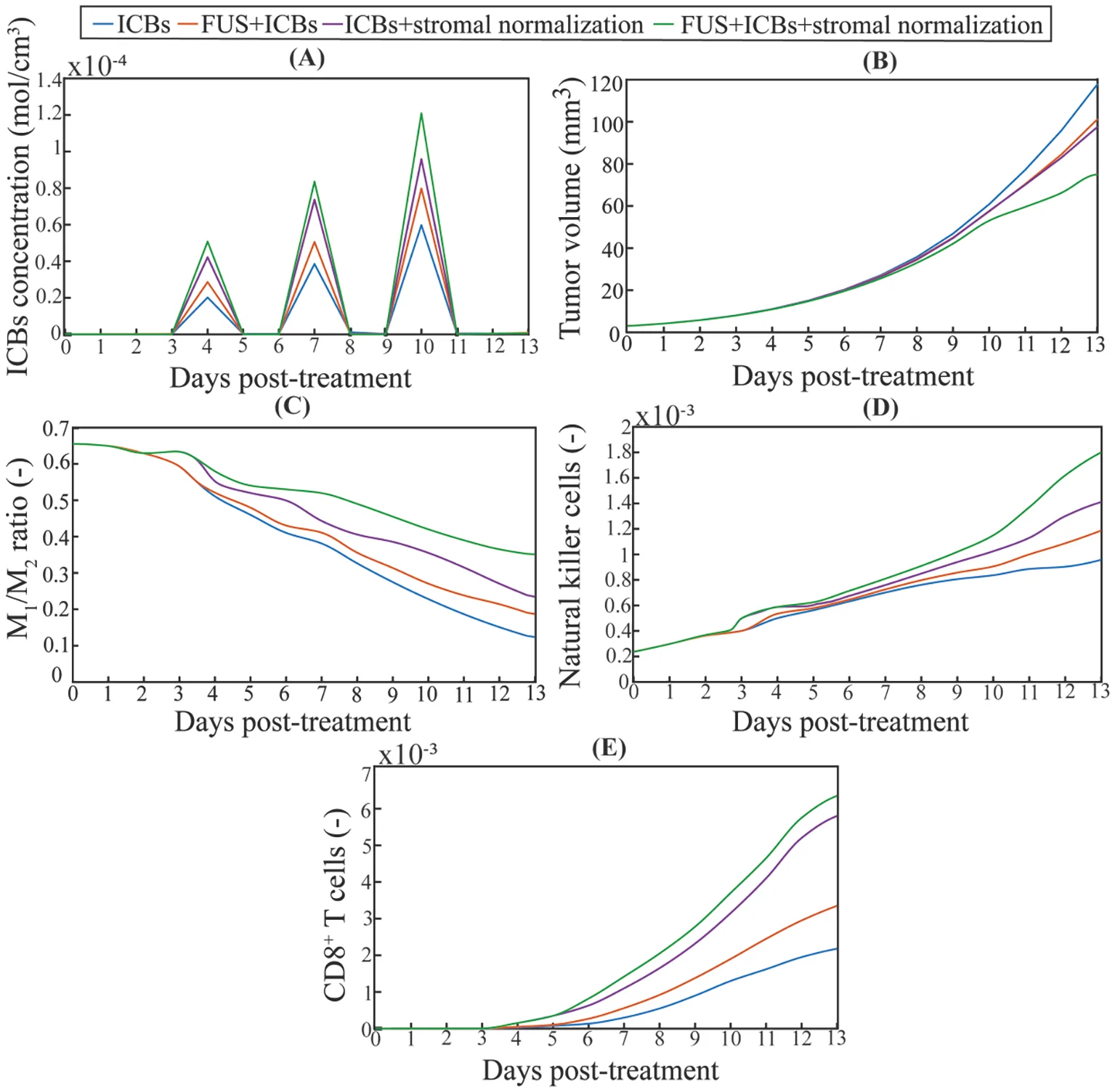
Effect of stromal normalization on the therapeutic efficacy. Model predictions comparing the effects of ICBs, FUS+ICBs, ICBs+stromal normalization and FUS+ICBs+stromal normalization on **(A)** intratumoral ICBs concentration, **(B)** tumor volume, **(C)** M_1_/M_2_ ratio, **(D)** natural killer cell population and **(E)** CD8^+^ T cell population.

An additional observation is that the immune cell populations predicted for the ICBs+stromal normalization and FUS+ICBs+stromal normalization groups begin to diverge from the corresponding groups without stromal normalization approximately on day 3 post-treatment, coinciding with the initiation of ICB administration (**Figures 4C–E**). This behavior is consistent with the experimentally observed delay required for stromal normalization agents to reduce tumor stiffness and normalize the stromal microenvironment. As tissue stiffness decreases and vascular functionality improves, immune cell trafficking into the tumor increases before the first administration of ICBs, creating a more favorable immune microenvironment for subsequent immunotherapy. These findings are consistent with previous experimental and computational studies demonstrating that stromal normalization enhances immune cell infiltration and improves the efficacy of immunotherapy (46, 92).

To complement the temporal analysis presented in **Figure 4**, the spatial distributions of ICBs concentration, M1/M2 macrophage ratio, natural killer cells and CD8^+^ T cells at post-treatment Day 10 are presented in **Figure 5**. The spatial distributions are consistent with the treatment-dependent trends observed in **Figure 4**, with ICBs+stromal normalization and FUS+ICBs+stromal normalization resulting in higher intratumoral ICBs concentrations and enhanced immune cell responses compared with ICBs and FUS+ICBs alone. These treatment strategies also result in a more uniform spatial distribution of the predicted responses within the tumor.

**Figure 5:**
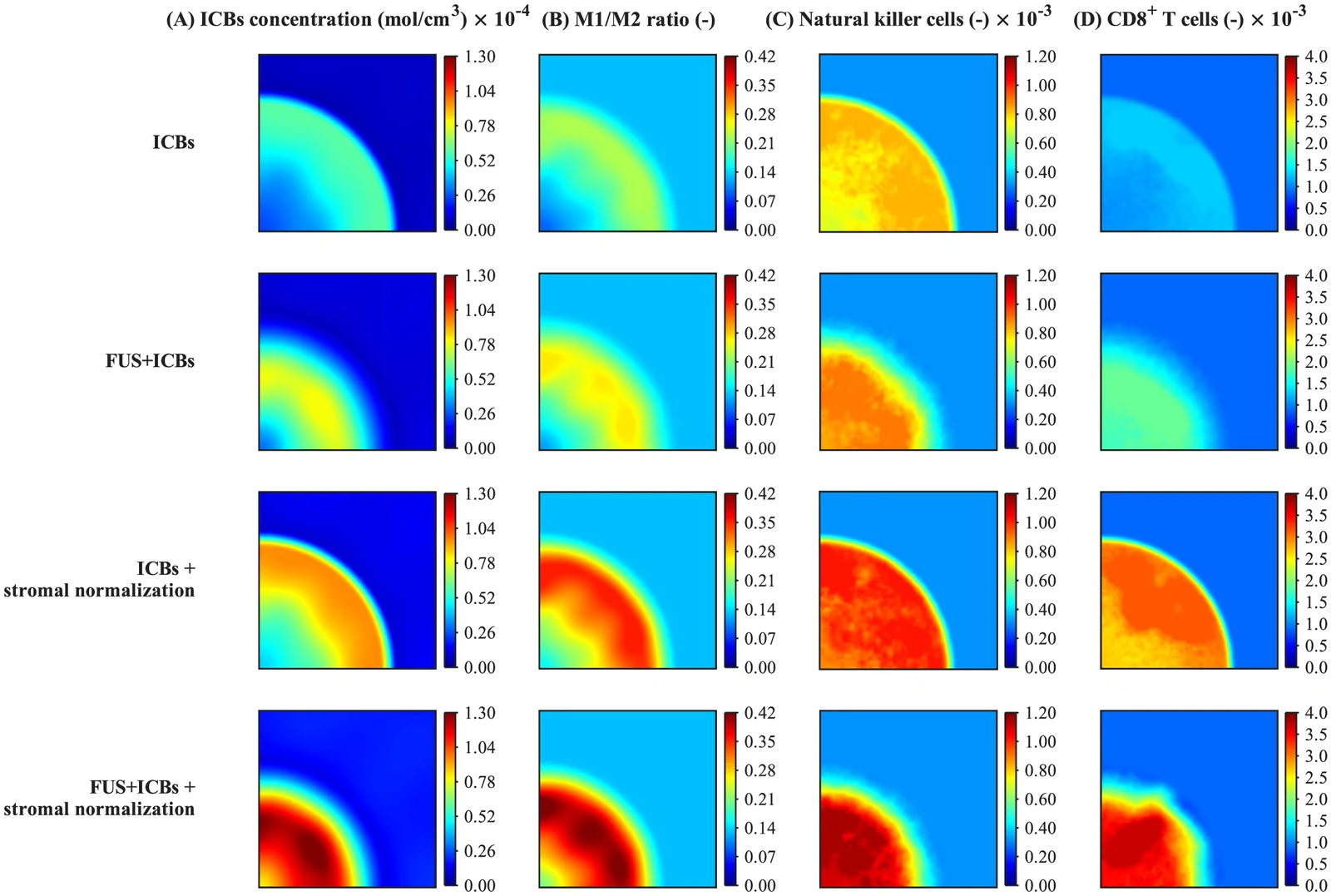
Spatial distributions of **(A)** ICBs concentration (mol/cm³), **(B)** M1/M2 ratio (-), **(C)** Natural killer cells (-) and **(D)** CD8^+^ T cells (-) for ICBs, FUS+ICBs, ICBs+stromal normalization and FUS+ICBs+stromal normalization, evaluated at post-treatment Day 10. The color bars indicate the magnitude of the corresponding variables.

To further investigate the influence of vascular permeability on treatment response, a parametric analysis was performed by varying the vessel wall pore radius (r_o_) from 25 nm to 200 nm, representing different degrees of vascular normalization and permeability enhancement (8, 107). The effects of r_o_ on intratumoral ICB concentration, tumor growth and immune cell populations are presented in **Figure 6**. As shown in **Figure 6A**, increasing the vessel wall pore radius progressively enhances the intratumoral accumulation of ICBs for both treatment scenarios (ICBs, solid lines and FUS+ICBs, dashed lines). The effect is considerably greater when FUS is combined with immunotherapy owing to the transient increase in vascular permeability induced by FUS. However, the curves corresponding to r_o_=100 nm and r_o_=200 nm are nearly identical, indicating that pore radius beyond approximately 100 nm provides only a marginal additional benefit in drug accumulation. This suggests that drug delivery reaches an optimum regime, where further enlargement of the vascular pores provides only a marginal additional increase in antibody transport into the tumor. The improved drug delivery is reflected in the tumor growth predictions (**Figure 6B**). Increasing r_o_ results in progressively greater tumor inhibition, with the largest reduction observed for the FUS+ICBs treatment at r_o_=100 nm and r_o_=200 nm. In contrast, for smaller pore radii (25–75 nm), the beneficial effect of FUS is more pronounced because the FUS-induced increase in vascular permeability provides a greater relative enhancement in drug transport than in tumors that already possess highly permeable vasculature.

**Figure 6:**
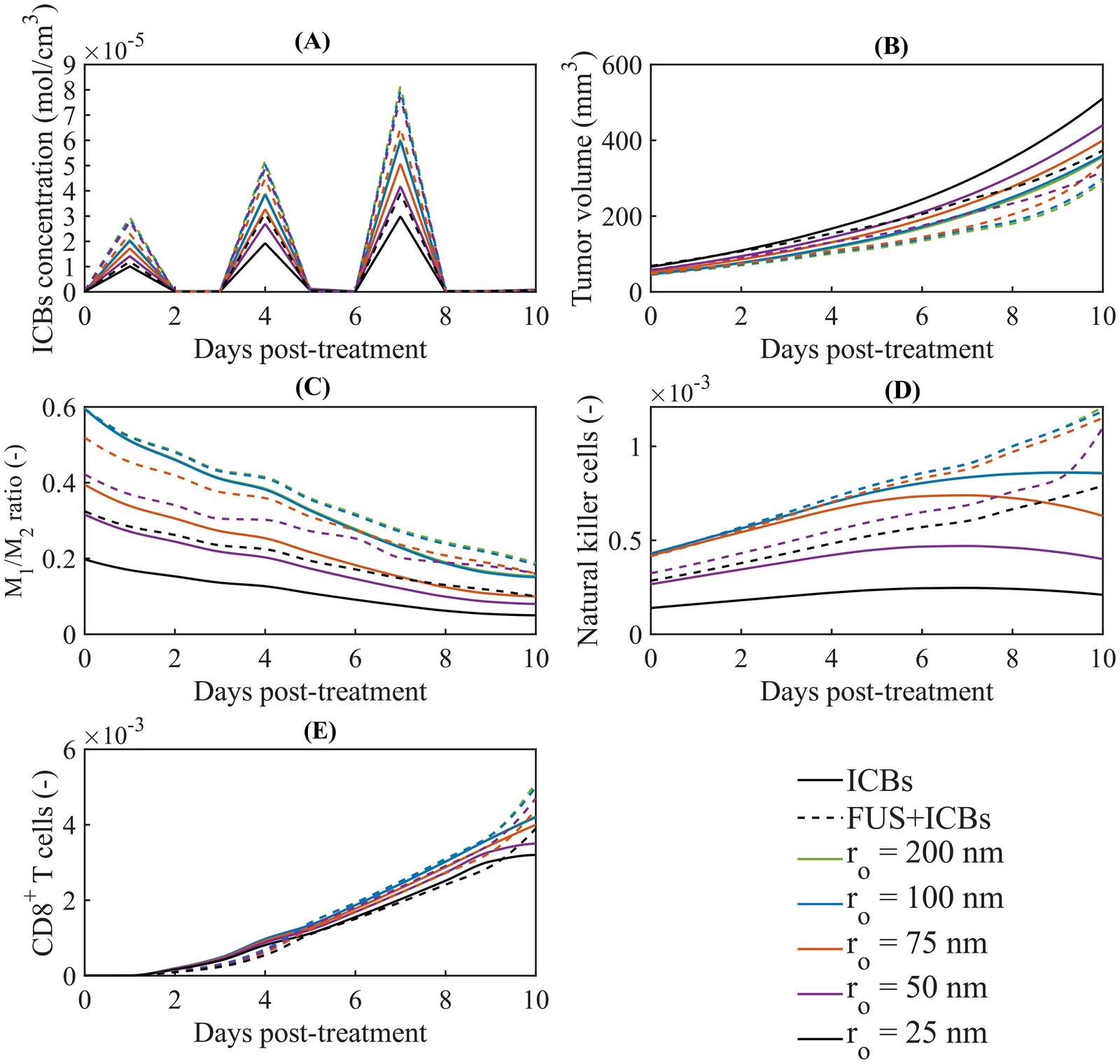
Effect of vessel wall pore radius (r_o_) on the therapeutic efficacy. Model predictions comparing the effects of ICBs and FUS+ICBs for different vessel wall pore radii (r_o_ = 25, 50, 75, 100 and 200 nm) on **(A)** intratumoral ICBs concentration, **(B)** tumor volume, **(C)** M_1_/M_2_ ratio, **(D)** natural killer cell population and **(E)** CD8^+^ T cell population.

The influence of vessel wall pore radius is also evident in the predicted immune response (**Figures 6C–E**). Increasing r_o_ promotes higher M_1_/M_2_ ratios, NK cell populations and CD8^+^ T cell populations, with the greatest responses consistently observed in the FUS+ICBs group. These findings indicate that improved vascular permeability not only enhances antibody delivery but also facilitates immune cell recruitment and promotes a more immunostimulatory TME. An additional observation concerns the timing of the immune response. The M_1_/M_2_ ratio and NK cell population exhibit distinct baseline values at the initiation of treatment (day 0 post-treatment) because each simulation is initialized with a different vessel wall pore radius (r_o_). Since r_o_ influences vascular permeability and immune cell trafficking throughout tumor development, differences in these innate immune populations are already established before treatment begins (**Figures 6C, D**). Following treatment initiation, these differences become progressively more pronounced as improved vascular transport further enhances immune cell recruitment. In contrast, CD8^+^ T cells (**Figure 6E**) display a delayed response because they belong to the adaptive immune system. Consequently, differences between the various pore-radius cases become apparent later in the treatment course. Overall, the model predicts that increasing vessel wall pore radius enhances both drug delivery and antitumor immune responses, thereby improving therapeutic efficacy. Nevertheless, the negligible differences observed between 100 nm and 200 nm suggest the existence of an upper limit beyond which further increases in vascular permeability yield diminishing therapeutic benefits.

To complement the temporal analysis presented in **Figure 6**, the spatial distributions of ICBs concentration, M1/M2 macrophage ratio, natural killer cells and CD8^+^ T cells at post-treatment Day 7 are presented in **Figure 7** respectively, for representative vessel wall pore radius of r_o_=25 nm and r_o_=200 nm. The spatial distributions are consistent with the trends observed in **Figure 6**, with the larger vessel wall pore radius resulting in higher intratumoral ICBs concentrations and enhanced immune cell responses. These effects are further enhanced when FUS is combined with ICBs, with the predicted responses exhibiting a more uniform spatial distribution within the tumor.

**Figure 7:**
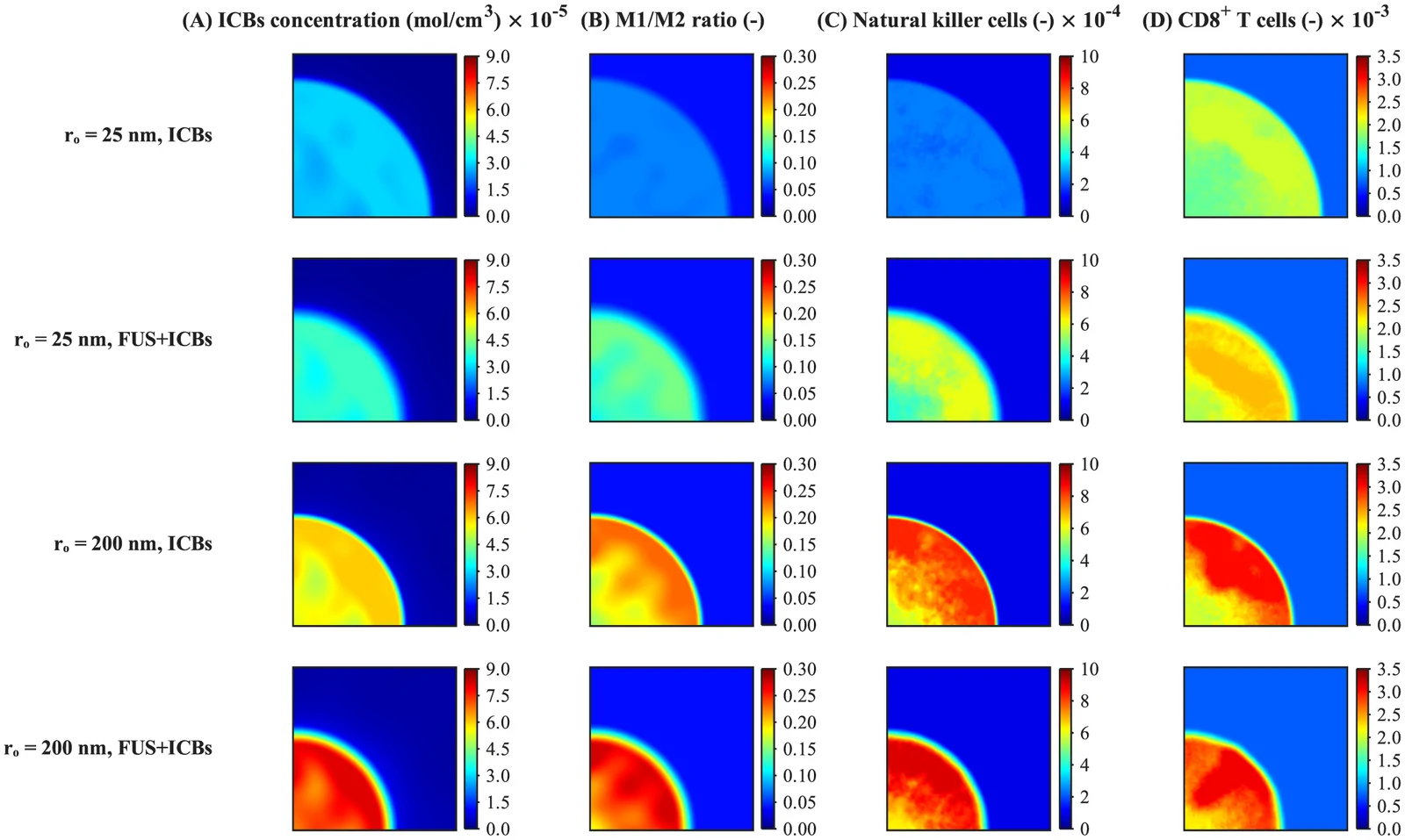
Spatial distributions of **(A)** ICBs concentration (mol/cm³), **(B)** M1/M2 ratio (-), **(C)** Natural killer cells (-) and **(D)** CD8^+^ T cells (-) for representative vessel wall pore radius of r_o_ = 25nm and r_o_ = 200nm, under ICBs and FUS+ICBs treatment, evaluated at post-treatment Day 7. The color bars indicate the magnitude of the corresponding variables.

The phase diagrams shown in **Figure 8** illustrate the combined effect of the tumor elastic modulus (E) and vessel wall pore radius (r_o_) on the therapeutic response to ICBs and FUS+ICBs. These diagrams illustrate the model predicted effects of tumor mechanical properties and vascular permeability jointly regulate drug delivery and treatment efficacy. For the ICBs treatment (**Figures 8A, B**), the lowest tumor volumes are predicted for tumors with low elastic modulus and large vessel wall pore radius, whereas the largest tumor volumes occur in tumors characterized by high stiffness and small vessel pores. This behavior reflects the combined effects of mechanical compression and limited vascular permeability. As the elastic modulus increases, compressed blood vessels reduce tissue perfusion, while smaller vessel pores further restrict antibody extravasation, thereby limiting intratumoral drug accumulation. Consequently, the PD-1/anti-PD-1 complex concentration (**Figure 8B**) follows the same dependence on the TME parameters. The highest complex concentrations are predicted for tumors with a low elastic modulus and a large vessel wall pore radius, where improved vascular functionality and permeability facilitate antibody transport into the tumor tissue. Interestingly, increasing only one of the two parameters is insufficient to maximize therapeutic efficacy. For example, although reducing the elastic modulus induces vascular decompression, the therapeutic response remains limited when the vessel wall pore radius is small because antibody transport across the vascular wall remains restricted.

**Figure 8:**
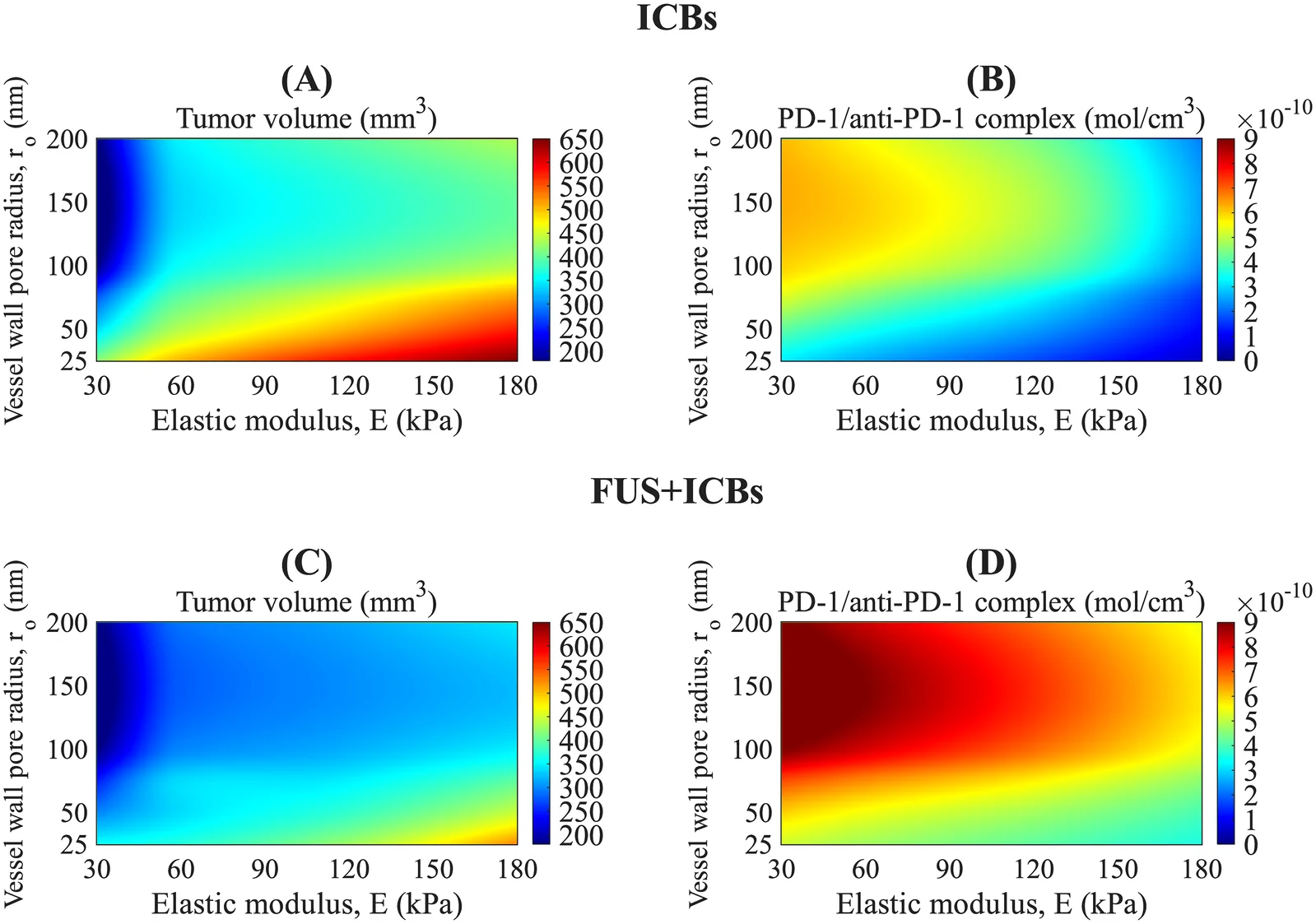
Phase diagrams illustrating the effects of TME parameters, including the vessel wall pore radius (r_o_) and tumor elastic modulus (E) on therapeutic response. **(A, B)** Tumor volume and anti-PD-1/PD-1 complex concentration, respectively, following ICBs treatment. **(C, D)** Corresponding predictions for FUS+ICBs treatment. The phase diagrams indicate how variations in tumor mechanical properties and vascular permeability influence drug delivery and treatment efficacy.

The beneficial effect of FUS is evident in **Figures 8C, D**. Compared with ICB monotherapy, FUS enlarges the region associated with favorable therapeutic outcomes across a broader range of elastic modulus and vessel pore radius values. Low tumor volumes and high PD-1/anti-PD-1 complex concentrations are achieved over a much broader range of intermediate elastic modulus and vessel pore radius values. For example, tumors with an elastic modulus of approximately 100 kPa and a vessel wall pore radius of approximately 50 nm, which exhibit only moderate responses to ICBs alone, demonstrate markedly improved therapeutic outcomes when FUS is incorporated. This improvement is attributed to the transient increase in vascular permeability induced by FUS, which enhances antibody transport even in tumors with partially compressed vasculature. Nevertheless, the phase diagrams also indicate the existence of a mechanical limit beyond which FUS alone cannot fully overcome the transport barriers imposed by the tumor microenvironment. In tumors characterized by both very high stiffness and very small vessel wall pore radius, therapeutic efficacy remains limited despite the application of FUS, as severely compressed blood vessels together with restricted pore size continue to impede efficient drug delivery. The mesh convergence of the pore-radius-dependent response was further assessed using a refined mesh, confirming mesh-independent results across the considered r_0_ range and consistent reproduction of the plateau observed at approximately r_o_=100 nm and above, as detailed in **Supplementary Figure S3** and **Supplementary Table S6**, **SI Appendix**. Overall, these results suggest that the therapeutic benefit of FUS is greatest for tumors with intermediate mechanical resistance, whereas optimal treatment efficacy is achieved when stromal normalization and increased vascular permeability act together to improve intratumoral antibody transport.

#### 3.2.2 Impact of ICBs parameters on treatment outcomes

The therapeutic efficacy of ICB is strongly influenced by the binding affinity between anti-PD-1 antibodies and PD-1 receptors expressed on immune cells (8, 57). To investigate this effect, a parametric analysis was performed by varying the anti-PD-1/PD-1 binding affinity, *μ*_PD-1/anti-PD-1_, over a broad range around the baseline value, from 127.821 to 1022.568 cm³/mol/day, where the baseline value used throughout the simulations was 511.284 cm³/mol/day (57). The results of the parametric analyses presented in this section represent model-derived predictions. For parameters incorporated through monotonic relationships in the governing equations, such as the anti-PD-1/PD-1 binding affinity, the direction of some predicted trends follows from the model formulation. The analyses quantify the resulting effects on drug delivery, checkpoint complex formation, tumor cell killing and tumor volume.

**Figure 9** summarizes the predicted effects of this parameter on drug transport, immune checkpoint inhibition and tumor progression. As shown in **Figure 9A**, increasing the binding affinity has a limited effect on the intratumoral concentration of anti-PD-1, since this quantity is primarily governed by drug transport rather than receptor binding. Nevertheless, the FUS+ICBs treatment consistently achieves higher intratumoral drug concentrations than ICB monotherapy owing to the transient increase in vascular permeability induced by FUS. In contrast, the effect of binding affinity becomes evident in the formation of the PD-1/anti-PD-1 complex (**Figure 9B**). Increasing *μ*_PD-1/anti-PD-1_ results in a monotonic increase in complex formation for both treatment groups, with the largest complex concentrations observed for the FUS+ICBs group. The higher antibody availability produced by FUS further promotes this effect, allowing a larger fraction of PD-1 receptors to become occupied by anti-PD-1 antibodies. The enhanced formation of the PD-1/anti-PD-1 complex is accompanied by a progressive reduction in the PD-1/PD-L1 complex (**Figure 9C**). As more PD-1 receptors become occupied by anti-PD-1 antibodies, fewer receptors remain available to interact with PD-L1 expressed by cancer cells. Consequently, the inhibitory immune checkpoint signaling is progressively suppressed, restoring the cytotoxic activity of immune cells against tumor cells (59).

**Figure 9:**
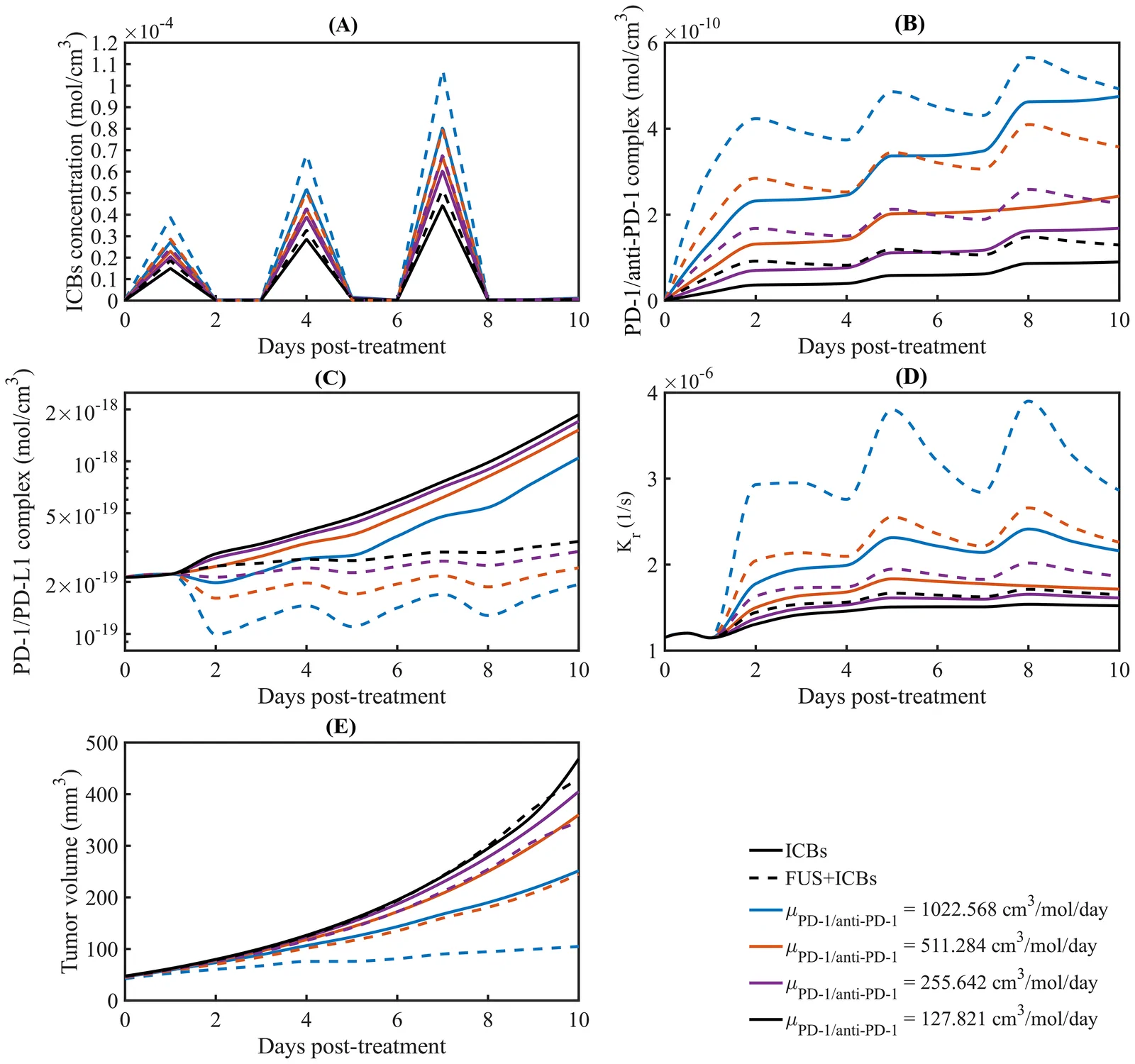
Effect of the binding affinity between PD-1 and anti-PD-1 complex (μ_PD-1/anti-PD-1_) on the therapeutic efficacy. Model predictions comparing the effects of ICBs and FUS+ICBs for different values of binding affinity (μ_PD-1/anti-PD-1_ = 127.821, 255.642, 511.284 and 1022.568 cm³/mol/day) on **(A)** intratumoral ICBs concentration, **(B)** PD-1/anti-PD-1 complex concentration, **(C)** PD-1/PD-L1 complex concentration, **(D)** tumor cell killing rate (K_r_) and **(E)** tumor volume.

This mechanism is directly reflected in the predicted cancer cell killing rate, K_r_ (**Figure 9D**). Here, K_r_ corresponds to the absolute value of the second term on the right-hand side of the cancer cell equation (Equation 8), which represents immune-mediated cancer cell elimination. In this formulation, increasing PD-1/PD-L1 complex formation progressively suppresses the cytotoxic activity of CD8^+^ T cells, NK cells and M_1_ macrophages, thereby reducing the effective cancer cell killing rate.

Therefore, the decrease in PD-1/PD-L1 complex concentration observed at higher binding affinities results in a corresponding increase in the effective cancer cell killing rate. The cumulative effects of these mechanisms are reflected in the predicted tumor growth (**Figure 9E**). Tumor volume decreases progressively with increasing anti-PD-1/PD-1 binding affinity for both treatment strategies, while the greatest therapeutic response is consistently obtained for the FUS+ICBs treatment combined with the highest binding affinity (1022.568 cm³/mol/day). These findings indicate that increasing the binding affinity enhances therapeutic efficacy by promoting antibody-receptor binding, reducing inhibitory PD-1/PD-L1 signaling, restoring immune cell cytotoxicity and ultimately increasing tumor cell elimination. Furthermore, the combination of FUS with ICBs provides an additional therapeutic advantage by improving intratumoral antibody delivery, thereby maximizing the biological benefits of high-affinity checkpoint inhibition.

To further evaluate the influence of anti-PD-1 availability on treatment response, a parametric analysis was performed by varying the anti-PD-1 source term, *γ*_anti-PD-1_, from 0.5 to 4 mol/cm^3^/day, with the baseline value set to 1 mol/cm^3^/day. This parameter represents the rate of anti-PD-1 administration in the model. The effects of *γ*_anti-PD-1_ on intratumoral antibody concentration, immune checkpoint complex formation, cancer cell killing and tumor progression are shown in **Figure 10**. As expected, increasing *γ*_anti-PD-1_ resulted in higher intratumoral ICB concentrations for both ICBs and FUS+ICBs treatment groups (**Figure 10A**). The highest antibody concentration was predicted for the FUS+ICBs group with *γ*_anti-PD-1_ = 4mol/cm^3^/day, reflecting the combined effect of increased antibody administration and FUS-enhanced vascular transport. This increase in antibody availability was directly reflected in the formation of the PD-1/anti-PD-1 complex (**Figure 10B**), which increased monotonically with increasing *γ*_anti-PD-1_. Consistent with the competitive binding mechanism included in the model, higher PD-1/anti-PD-1 complex formation was associated with a reduction in the PD-1/PD-L1 complex (**Figure 10C**). As more PD-1 receptors were occupied by anti-PD-1 antibodies, fewer PD-1 receptors remained available to bind PD-L1 expressed by cancer cells, thereby reducing inhibitory checkpoint signaling. This reduction in PD-1/PD-L1-mediated inhibition led to an increase in the effective cancer cell killing rate, K_r_, particularly in the FUS+ICBs group (**Figure 10D**).

**Figure 10:**
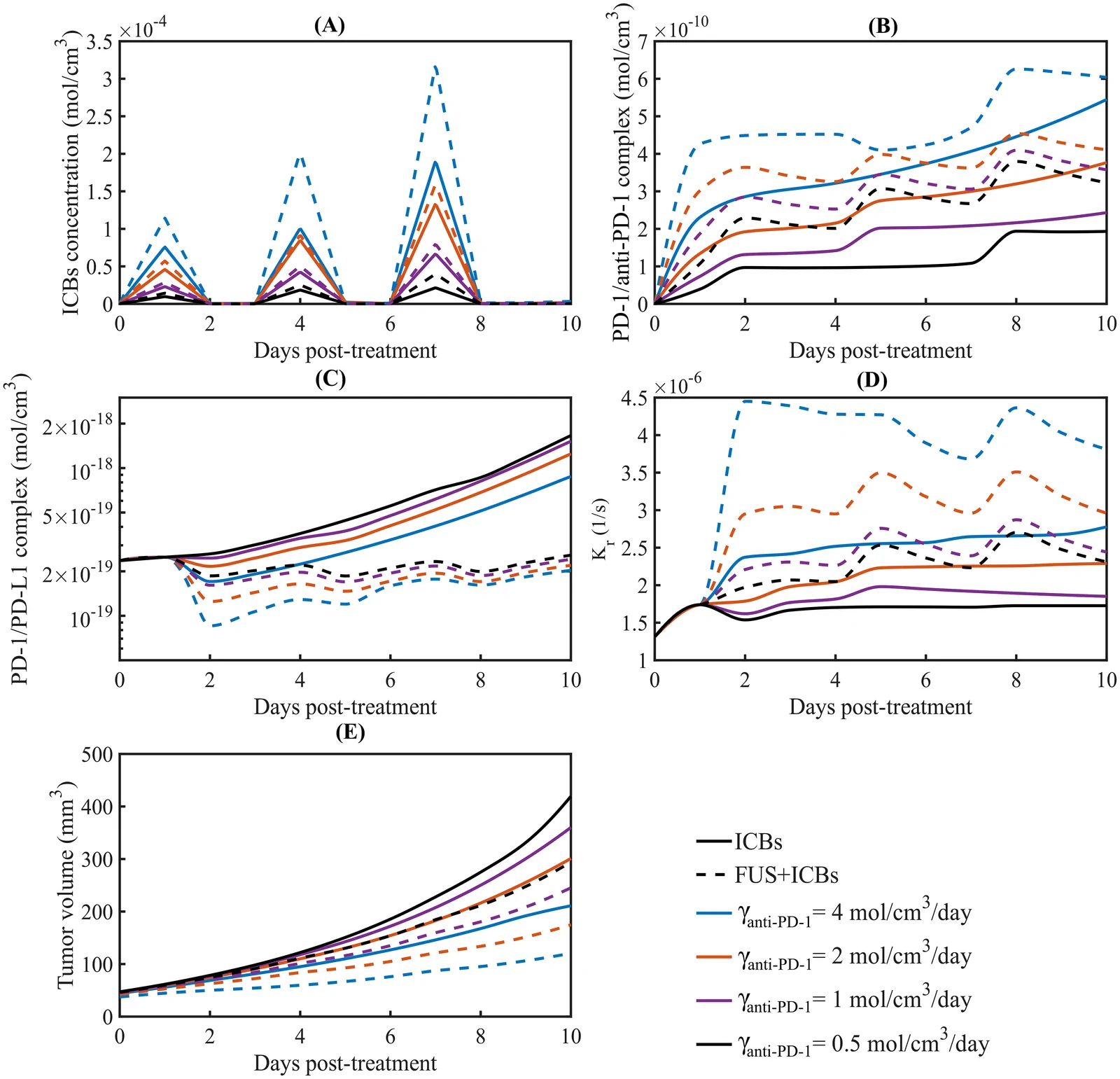
Effect of the ICBs source term (γ_anti-PD-1_) on the therapeutic efficacy. Model predictions comparing the effects of ICBs and FUS+ICBs for different values of anti-PD-1 source term (γ_anti-PD-1_ = 0.5, 1, 2 and 4 mol/cm³/day) on **(A)** intratumoral ICBs concentration, **(B)** PD-1/anti-PD-1 complex concentration, **(C)** PD-1/PD-L1 complex concentration, **(D)** tumor cell killing rate (K_r_) and **(E)** tumor volume.

The combined effects of increased antibody delivery, enhanced PD-1 blockade and improved immune-mediated killing resulted in progressively stronger tumor suppression as *γ*_anti-PD-1_ increased (**Figure 10E**). The greatest tumor volume reduction was observed for the FUS+ICBs treatment with *γ*_anti-PD-1_ = 4 mol/cm^3^/day. An additional observation is that the relative benefit of FUS is more evident at lower anti-PD-1 source values, where antibody availability is more limited. At higher *γ*_anti-PD-1_, the differences between ICBs and FUS+ICBs become less pronounced, suggesting that increasing antibody administration partially compensates for transport limitations. Overall, these results indicate that anti-PD-1 availability is a key determinant of therapeutic efficacy, while FUS provides an additional advantage by improving antibody transport, particularly when the administered antibody source is limited.

Finally, a combined parametric analysis was performed to investigate the simultaneous effects of the anti-PD-1/PD-1 binding affinity (*μ*_PD-1/anti-PD-1_) and the anti-PD-1 source term (*γ*_anti-PD-1_) on therapeutic response. The corresponding phase diagrams are presented in **Figure 11**, where the binding affinity varies from 127.821 to 1022.568 cm^3^/mol/day, while the anti-PD-1 source term ranges from 0.5 to 4 mol/cm^3^/day. The phase diagrams illustrate the predicted tumor volume and PD-1/anti-PD-1 complex concentration for both ICBs (**Figures 11A, B**) and FUS+ICBs (**Figures 11C, D**). For ICB monotherapy, increasing either the binding affinity or the administered anti-PD-1 source progressively improved the therapeutic response (**Figure 11A**). The greatest tumor inhibition was achieved when both parameters simultaneously attained their highest values, whereas poor therapeutic efficacy was predicted for low binding affinity combined with limited antibody administration. A similar trend was observed for the PD-1/anti-PD-1 complex (**Figure 11B**), where increasing both parameters promoted greater receptor occupancy by anti-PD-1 antibodies, indicating more effective immune checkpoint blockade. The beneficial effect of FUS becomes more evident in **Figures 11C, D**. Compared with ICB monotherapy, the region associated with favorable therapeutic outcomes extends toward intermediate values of both parameters. Consequently, tumor reduction is predicted even for moderate anti-PD-1 source values (approximately 2 mol/cm^3^/day) and intermediate binding affinities (approximately 500 cm^3^/mol/day), suggesting that FUS partially compensates for less favorable antibody properties by enhancing intratumoral drug transport. The same behavior is reflected in the PD-1/anti-PD-1 complex (**Figure 11D**), where high complex concentrations are maintained over a considerably broader range of parameter combinations than for ICB monotherapy.

**Figure 11:**
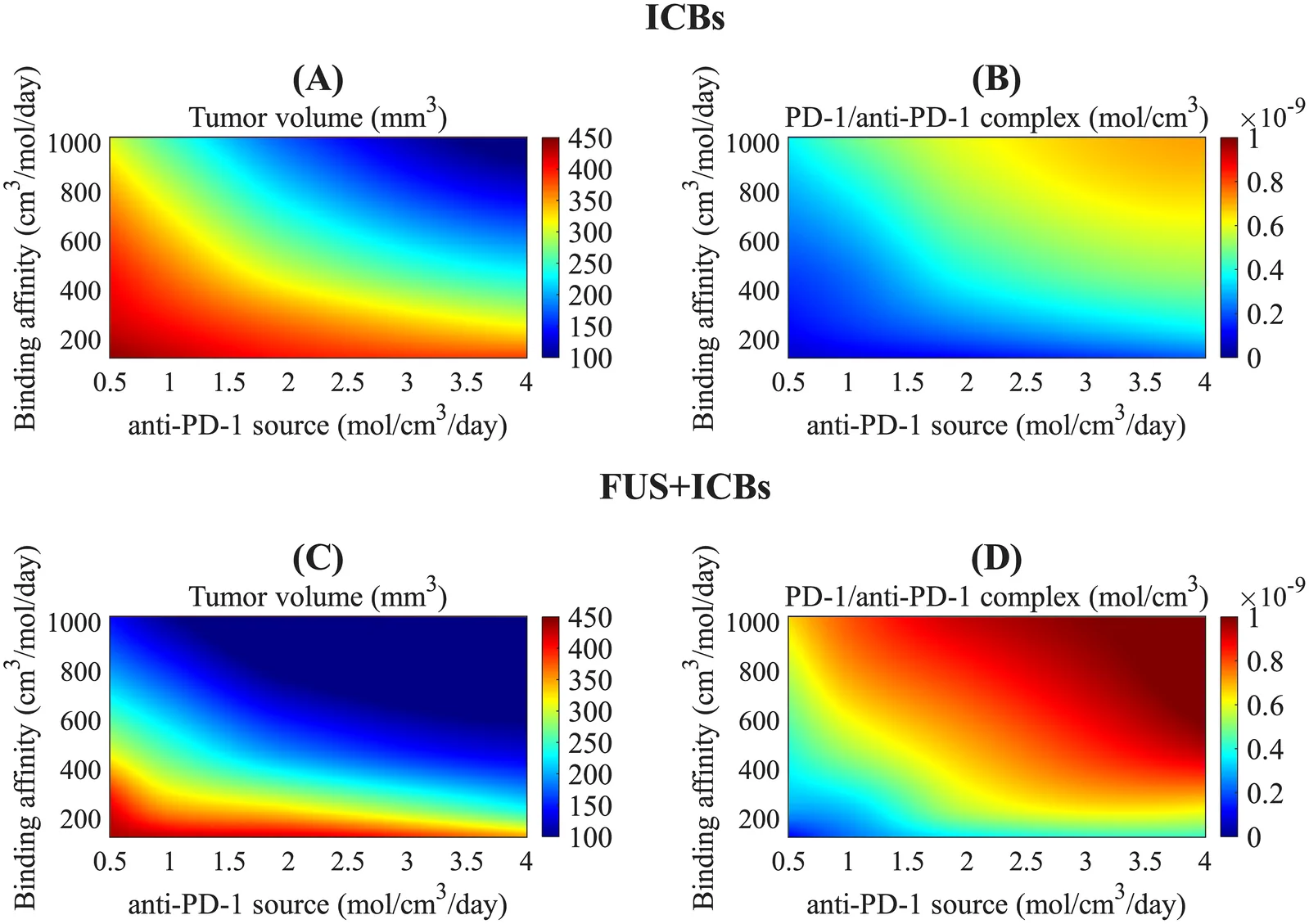
Phase diagrams illustrate the effects of drug-related parameters, including the binding affinity between PD-1 and anti-PD-1 (μ_PD-1/anti-PD-1_) and the anti-PD-1 source term (γ_ant-PD-1_) on therapeutic response. **(A, B)** Tumor volume and PD-1/anti-PD-1 complex concentration, respectively, following ICBs treatment. **(C, D)** Corresponding predictions for FUS+ICBs treatment. The phase diagrams indicate how variations in anti-PD-1 delivery and PD-1/anti-PD-1 binding affinity influence drug-target interactions and treatment efficacy.

However, the phase diagrams also indicate that FUS cannot fully overcome the limitations associated with simultaneously low antibody administration and weak binding affinity. When both the anti-PD-1 source and binding affinity remain low (e.g., 0.5 mol/cm^3^/day and approximately 200 cm^3^/mol/day, respectively), only modest improvements are observed following FUS treatment. These findings suggest that although FUS substantially broadens the range of effective therapeutic conditions, adequate antibody availability and sufficient binding affinity remain essential for achieving optimal immune checkpoint blockade. Overall, the model predictions indicate that therapeutic efficacy depends on the interplay between antibody pharmacodynamics and drug delivery. While increasing either parameter individually improves treatment outcome, the greatest benefit is achieved when high anti-PD-1 availability is combined with strong receptor binding. Furthermore, FUS enlarges the parameter space associated with effective therapy, highlighting its potential to improve treatment outcomes even when antibody characteristics are not optimal. The mesh convergence of the binding-affinity-dependent response was further assessed using a refined mesh, confirming mesh-independent results across the considered binding affinity range and consistent reproduction of the predicted changes in tumor volume and PD-1/anti-PD-1 complex concentration, as detailed in **Supplementary Figure S4** and **Supplementary Table S7**, **SI Appendix**.

## 4 Discussion

The present study developed a three-dimensional multiphysics computational framework to investigate how FUS-mediated modulation of the TME enhances the therapeutic efficacy of ICBs in solid tumors. Building upon our previously developed and validated mathematical framework (8, 46, 57, 61–63, 67, 68, 92, 93, 108) the proposed model integrates FUS-induced modulation of tumor transport, anti-PD-1 pharmacodynamics and innate and adaptive immune responses within a unified tissue-level framework. This enables mechanistic investigation of how tumor biomechanics, transport phenomena and antibody pharmacological characteristics collectively regulate therapeutic efficacy. Furthermore, the parametric analyses presented herein identify the key tumor and treatment-related parameters governing ICB efficacy and define the conditions under which FUS provides the greatest therapeutic benefit.

The predictive capability of the proposed framework was validated against two independent *in vivo* studies involving C6 glioma and 4T1 murine breast tumors (27, 37). The model accurately reproduced tumor growth together with the associated adaptive and innate immune responses following ICBs and FUS+ICBs therapy. Notably, for validation against each independent *in vivo* dataset, only the tumor-specific proliferation parameter k_1_ was calibrated, while all other model parameters were kept fixed at values obtained from previously calibrated models in the literature or determined during the initial development of the present framework. This minimal calibration strategy supports the predictive capability of the proposed framework across biologically distinct tumor types. Although the model reproduced the direction of the CD4^+^ T cell response in both validation datasets, it overpredicted its magnitude in C6 glioma and underpredicted it in 4T1 tumors, indicating that tumor specific differences in the magnitude of the CD4^+^ T cell response are not fully captured by the current model formulation.

The parametric analysis highlighted the importance of the physical characteristics of the TME in determining ICB efficacy. Stromal normalization emerged as one of the most effective strategies by reducing tumor stiffness, improving vascular function and enhancing antibody transport and immune cell infiltration. Consequently, combining stromal normalization with FUS and ICBs produced the greatest therapeutic benefit. An important observation was that the effects of stromal normalization became apparent approximately three days after treatment initiation, consistent with the experimentally reported delay required for mechanotherapeutics to remodel the stromal microenvironment (104, 106). These findings further support stromal normalization as a promising strategy for improving immunotherapy by alleviating the physical barriers imposed by the TME (46, 92). The simulations further indicated that vascular permeability is another key determinant of treatment efficacy. Increasing the vessel wall pore radius enhanced antibody delivery and tumor control, although the therapeutic benefit approached a plateau at larger pore sizes, suggesting that additional transport limitations become dominant. Phase diagram analysis further showed that FUS broadens the range of tumor mechanical conditions associated with favorable therapeutic outcomes, particularly for tumors with intermediate stiffness and vascular permeability. However, FUS alone could not overcome the severe transport limitations of highly compressed tumors with very small vessel pores.

Besides the physical characteristics of the TME, the pharmacological properties of ICBs also strongly influenced therapeutic efficacy. Increasing either the anti-PD-1/PD-1 binding affinity or the administered antibody dose enhanced PD-1 blockade, reduced PD-1/PD-L1 complex formation and increased immune-mediated tumor killing, resulting in greater tumor suppression. These findings are consistent with the established mechanism of PD-1 blockade, whereby anti-PD-1 antibodies inhibit PD-1/PD-L1 interactions and restore anti-tumor immunity (59). The simulations further showed that the therapeutic benefit of FUS depends on the pharmacological characteristics of the administered antibody. FUS provided the greatest benefit when antibody binding affinity or administration dose was limited by enhancing intratumoral antibody delivery and expanding the range of conditions under which effective ICB could be achieved. In contrast, its additional benefit diminished as receptor occupancy approached saturation. Overall, these findings indicate that favorable therapeutic outcomes are associated with the combined modulation of antibody pharmacological characteristics and tumor transport properties.

Certain limitations in the modeling framework should be recognized. First, the framework assumes spatially uniform tissue properties, omits vessel-level heterogeneity and treats FUS-induced transport enhancements as spatially uniform and temporally prescribed. Introducing discrete vascular networks could improve spatial resolution and enhance the accuracy of local drug distribution predictions. Furthermore, integrating image-derived tumor geometries that reflect the heterogeneous microvascular architecture would yield a more physiologically realistic depiction of solid tumors. Such refinements would enable a deeper exploration of how vascular structures modulate the interplay between FUS-induced tumor transport enhancement and ICBs delivery. Image-based modeling could also pave the way for patient-specific simulations that capture individual tumor characteristics, thereby supporting the development of personalized therapeutic strategies (55, 109, 110).

The current model focuses on the effects of FUS on the transport properties of the TME, which constitute the primary mechanism governing anti-PD-1 delivery within the proposed mathematical framework. In the experimental studies considered for model validation, FUS was applied in combination with microbubbles to transiently increase vascular permeability and facilitate antibody delivery (37). In the present model, these biological effects are incorporated through experimentally supported increases in vessel wall porosity, functional vascular density and interstitial hydraulic conductivity, without explicitly resolving the underlying acoustic-microbubble interactions. Accordingly, the FUS component of the present framework represents a phenomenological ultrasound-response model, in which the effects of FUS are incorporated through experimentally supported, time-dependent changes in tumor transport properties rather than through explicit simulation of the acoustic field. Because acoustic exposure parameters, including pressure, intensity, frequency, mechanical index, pulse duration and duty cycle, are not directly coupled to the resulting changes in tissue transport properties, the current framework cannot determine or optimize the acoustic exposure conditions required to produce these effects. Although this approach captures the overall enhancement of antibody transport observed experimentally, it does not distinguish the relative contributions of stable cavitation, inertial cavitation (111, 112) or other ultrasound-induced mechanical phenomena. Incorporating explicit models of bubble dynamics and cavitation physics would therefore provide a more mechanistic description of FUS-mediated vascular modulation and could further improve the predictive capability of the computational framework (113, 114).

The present framework was developed to investigate FUS in combination with ICB therapy, whereas effects of FUS are represented through changes in tumor transport properties, including vascular permeability, vessel wall porosity, functional vascular density and interstitial hydraulic conductivity. A FUS-alone treatment group was not included in the validation or parametric analyses. Although the experimental studies used for validation reported additional effects of FUS on the TME, the mechanisms linking these effects to changes in tumor growth and individual immune cell populations were not sufficiently characterized to be incorporated into the model. Therefore, the effects of FUS as a standalone treatment were not evaluated.

An additional limitation of the present framework is the use of a 1/8 domain under an idealized geometric and physical symmetry assumption, whereby all modeled components are imposed in a symmetry-consistent manner. Implementing 3D, image-based geometries could therefore improve the translational and predictive value of our computational framework. Finally, tumor tissue was modeled as a poroelastic medium (115–117), where the solid matrix is treated as purely elastic and interstitial fluid transport follows Darcy’s law. Nevertheless, these assumptions are expected to influence the model predictions primarily in a quantitative rather than a qualitative manner. The principal mechanistic conclusions remain robust, demonstrating that FUS-induced modulation of tumor transport properties acts in combination with ICB by enhancing antibody delivery, promoting immune cell infiltration and improving anti-tumor immunity within the TME. Overall, the proposed framework provides a comprehensive computational platform for investigating and optimizing FUS-assisted ICB and identifying treatment strategies that enhance the combined effects of TME normalization and cancer immunotherapy.

## Data Availability

The datasets presented in this study can be found in online repositories. The names of the repository/repositories and accession number(s) can be found in the article/**Supplementary Material**.
