# Supplementary material for: A predictive multiphysics framework for optimizing focused ultrasound-assisted immune checkpoint blockade
Source: Front Immunol. 2026 Oct 7;17:1937764. doi: 10.3389/fimmu.2026.1937764 (PMC13683793; doi:10.3389/fimmu.2026.1937764)
Supplement: Supplementary file 1 [file DataSheet1.pdf]

A predictive multiphysics framework for optimizing focused ultrasound-assisted immune checkpoint blockade

1 Supplementary Data

Mathematical model formulation

Kinematic principles underlying tumor growth

Equations (S1-S3) and (S20-S28) establish the comprehensive framework for the biomechanical model of tumor growth, which is solved to simulate the progression of the tumor within normal/host tissue. The solutions to these equations facilitate the computation of interstitial fluid pressure, fluid velocity, and the stresses generated through mechanical interactions between the tumor and the host tissue. Interstitial fluid pressure and fluid velocity are incorporated into the drug transport equation (Equation 5, in the main text), while the mechanical interactions between the tumor and the host tissue influence the tumor's growth rate. Equations (S4-S14) serve to clarify the balance law concerning the various cell types within the tumor to measure the growth stretch ratio, λ_g, which determines the rate of tumor proliferation. This growth stretch ratio is utilized within the general framework via Equations (S2, S3, and S17). All parameter values of this mathematical model are presented in Tables S1 and S2. This mathematical framework employs a robust systems-biology approach, which is meticulously designed to incorporate the intricate cellular and subcellular phenomena that occur within biological systems, specifically utilizing a continuum mechanics and finite elements model to thoroughly analyze the multifaceted dynamics of tumor growth. Also, Table S3 illustrates the initial values of the variables used in the mathematical model at time t=0 day.

In order to describe the kinematic behavior of the tumor, the multiplicative decomposition of the deformation gradient tensor is utilized. The tensor $\mathbf{F}$ is divided into two distinct components: the tumor's growth, denoted as $\mathbf{F}_g$ and the elastic mechanical interaction, represented as $\mathbf{F}_e$ (Ambrosi and Mollica 2002; Mpekris et al. 2015; Rodriguez, Hoger, and McCulloch 1994; Skalak et al. 1996):

$$\mathbf{F} = \mathbf{F}_e \mathbf{F}_g \tag{S1}$$

Henceforth, all tensor representations will be rendered in boldface to facilitate their distinction from other terms. It is important to recognize that, given the assumptions inherent in the model and the specific problem under investigation, the deformation gradient tensor can be expressed as a combination of more than two independent components, which separately encompass growth, residual stress formation, or extracellular matrix (ECM) remodeling (Skalak et al. 1996; Stylianopoulos and Jain 2013; Mascheroni et al. 2018). The growth component, denoted as $\mathbf{F}_g$, has been characterized as isotropic (its properties are the same in all directions) and homogeneous (it is uniform in composition

and structure throughout the entire volume), representing a non-stress inducing deformation gradient tensor that represents the proliferation of cancer cells (Kim, Stolarska, and Othmer 2011; Roose et al. 2003; Stylianopoulos et al. 2013; Pirentis et al. 2015).

$$\mathbf{F}_g = \lambda_g \mathbf{I} \tag{S2}$$

where λ_g is the growth stretch ratio, which assesses the proliferation of cancer cells, while **I** represents the second-order identity tensor, indicating that the proliferation associated with tumor growth occurs uniformly and isotropically. The elastic component, $\mathbf{F}_e$, accounts for the stress-inducing elastic interactions both within the tumor and with the surrounding host tissue. The elastic component of **F** was derived directly from Equation (S1) as:

$$\mathbf{F}_e = \mathbf{F}\,\mathbf{F}_g^{-1} \tag{S3}$$

Calculation of the growth stretch ratio λ_g

The rate of tumor growth is measured by taking into account both the concentration of oxygen and the proliferation of cancer cells (Voutouri and Stylianopoulos 2014; Kim, Stolarska, and Othmer 2011; MacLaurin et al. 2012; Mpekris et al. 2018; Roose et al. 2003). The equation utilized is:

$$\frac{d\lambda_g}{dt} = \frac{1}{3}(\mathrm{CCs} \cdot \mathrm{S}_{\mathrm{CCs}}) \cdot \lambda_g \tag{S4}$$

where CCs represents the population of non-stem-like cancer cells (CCs). The $\mathrm{S}_{\mathrm{CCs}}$ is the respective rate of proliferation and degradation for the CCs, as specified subsequently in Equation (16).

Implementation of biphasic theory for understanding the mechanical behavior of the tumor

The conservation principles of both the solid and fluid phases within the tumor system are expressed through the subsequent mass balance equations: (Roose et al. 2003; Voutouri and Stylianopoulos 2014)

$$\frac{\partial \boldsymbol{\Phi}^c}{\partial t} + \nabla \cdot (\boldsymbol{v}^s \boldsymbol{\Phi}^c) = \mathrm{CCs} \cdot \mathrm{S}_{\mathrm{CCs}} \tag{S5}$$

$$\frac{\partial \boldsymbol{\Phi}^f}{\partial t} + \nabla \cdot (\boldsymbol{v}^f \boldsymbol{\Phi}^f) = \mathrm{Q} \tag{S6}$$

In this context, $\boldsymbol{\Phi}^{c}$ and $\boldsymbol{\Phi}^{f}$ represent the volume fractions of the solid and fluid phases, respectively, while $\boldsymbol{v}^{s}$ and $\boldsymbol{v}^{f}$ denote their associated velocities.

The variable Q in Equation (S6) signifies the fluid flux entering the tumor from blood vessels as well as from the adjacent normal tissue, subtracted by the fluid flux exiting via lymphatic vessels and is expressed as (Stylianopoulos et al. 2013):

$$\mathrm{Q} = \mathrm{L_p} \cdot \mathrm{S_v} \cdot (\boldsymbol{p}_{\mathrm{v}} - \boldsymbol{p}_{\mathrm{i}}) - \mathrm{L_{pl}} \cdot \mathrm{S_{vl}} \cdot (\boldsymbol{p}_{\mathrm{i}} - \boldsymbol{p}_{\mathrm{l}}) \tag{S7}$$

where $\mathrm{L_p}$, $\mathrm{S_v}$ and $\boldsymbol{p}_{\mathrm{v}}$ are indicative of the hydraulic conductivity, vascular density and vascular pressure, respectively; $\mathrm{L_{pl}}$, $\mathrm{S_{vl}}$ and $\boldsymbol{p}_{\mathrm{l}}$ are the analogous parameters for lymphatic vessels; and $\boldsymbol{p}_{\mathrm{i}}$ corresponds to the interstitial fluid pressure. In this particular model configuration and taking into account the principle of mass conservation within the tissue, the total of the solid and fluid volume fractions is established to consistently amount to one, in accordance with Equation (S6).

$$\boldsymbol{\Phi}^{\mathrm{f}} = 1 - \boldsymbol{\Phi}^{\mathrm{c}} \tag{S8}$$

Moreover, summing Equations (S5) and (S6), the mass balance can be expressed as follows:

$$\nabla \cdot \left(\boldsymbol{\Phi}^{\mathrm{c}} \boldsymbol{v}^{\mathrm{s}} + \boldsymbol{\Phi}^{\mathrm{f}} \boldsymbol{v}^{\mathrm{f}} \right) = \mathrm{Q} + \mathrm{CCs} \cdot \mathrm{S_{CCs}} \tag{S9}$$

where the fluid velocity $\boldsymbol{v}^{f}$ is defined according to Darcy's law (Byrne and Preziosi 2003)

$$\boldsymbol{\Phi}^{\mathrm{f}} (\boldsymbol{v}^{\mathrm{f}} - \boldsymbol{v}^{\mathrm{s}}) = -\mathrm{k_{th}} \cdot \nabla \boldsymbol{p}_{\mathrm{i}} \Rightarrow \boldsymbol{v}^{\mathrm{f}} = -\frac{\mathrm{k_{th}}}{\boldsymbol{\Phi}^{\mathrm{f}}} \nabla \boldsymbol{p}_{\mathrm{i}} + \boldsymbol{v}^{\mathrm{s}} \tag{S10}$$

with $\mathrm{k_{th}}$ denoting the hydraulic conductivity of the interstitial medium (Stylianopoulos et al. 2008).

In accordance with the biphasic theory applicable to soft tissues (Mow et al. 1980), the overall stress tensor $\boldsymbol{\sigma}_{\mathrm{tot}}$ is constituted by the fluid phase stress tensor $\boldsymbol{\sigma}^{\mathrm{f}} = -\boldsymbol{p}_{\mathrm{i}}\mathbf{I}$ and the solid phase stress tensor $\boldsymbol{\sigma}^{\mathrm{s}}$. Consequently, the stress balance can be expressed as:

$$\nabla \cdot \boldsymbol{\sigma}_{\mathrm{tot}} = \mathbf{0} \Rightarrow \nabla \cdot (\boldsymbol{\sigma}^{\mathrm{s}} - \boldsymbol{p}_{\mathrm{i}}\mathbf{I}) = \mathbf{0} \tag{S11}$$

where the Cauchy stress tensor associated with the solid phase $\boldsymbol{\sigma}^{\mathrm{s}}$ is delineated by: (Taber 2008)

$$\boldsymbol{\sigma}^{s} = \mathbf{J}_{e}^{-1}\mathbf{F}_{e}\frac{\partial W}{\partial \mathbf{F}_{e}^{T}}, \tag{S12}$$

The mechanical properties of the tumor have been defined as incompressible and neo-Hookean, with the strain energy density represented by: (Ciarletta 2013; Voutouri et al. 2014; Xu, Bayly, and Taber 2009; Xu et al. 2010)

$$W = \frac{1}{2}\mu\left(-3 + II_{1}\right) - \boldsymbol{p}\left(\mathbf{J}_{e} - I_{1}\right) - \left(\frac{p^{2}}{2k}\right) \tag{S13}$$

where μ and k denote the shear and bulk modulus of the material, respectively; J_e signifies the determinant of the elastic deformation gradient tensor $\mathbf{F}_e$; $II_1 = I_1 \boldsymbol{J}_e^{-2/3}$ with $I_1 = \text{tr}\mathbf{C}_e$ being the first invariant of the elastic Cauchy-Green deformation tensor $\mathbf{C}_e = \mathbf{F}_e^{T}\mathbf{F}_e$, and p is a penalty variable introduced for materials that exhibit near incompressibility that regularizes the constraint involved in the second term (Holzapfel, Gasser, and Ogden 2000). Values of shear modulus are based on experimental findings from our previous studies (Mpekris et al. 2024; Panagi et al. 2024). The adjacent normal tissue is presumed to be compressible and neo-Hookean, characterized by a Poisson ratio of 0.2 and tumor tissue with a Poisson ratio of 0.45.

Functional vascular density

To assess the functional vascular density, it is claimed that is impacted by the decrease in the diameter of blood vessels (d/d_0) linked to the increase of solid stress (Mpekris et al. 2015). Furthermore, the functional vascular density is dependent on the permeability of the tumor vascular wall (Stylianopoulos and Jain 2013), as hyper-permeable vessels diminish both perfusion and overall functionality of the vessels. The functional vascular density can be expressed as:

$$S_{v} = \frac{d}{d_{0}} S_{v}^{0} \rho_{v}^{EC}, \tag{S14}$$

where S_v^0 will be determined by the size of the pores in the vascular wall (i.e., its permeability) and ρ_v^{EC} is related to the density of endothelial cells.

Transport of oxygen

The variation in oxygen levels within the tumor tissue is considered to be based on both its transport mechanisms, namely convection and diffusion, alongside the rate of oxygen utilized by the cells, as well as the influx of oxygen from the surrounding blood vessels (Kim, Stolarska, and Othmer 2011; Roose et al. 2003), specifically described by the equation:

$$\frac{\partial c_{ox}}{\partial t} + \nabla \cdot \left(c_{ox} \boldsymbol{v}^f \right) = D_{ox} \cdot \nabla^2 c_{ox} - \frac{A_{ox} \cdot c_{ox}}{c_{ox} + k_{ox}} \cdot CCs + P_{er} \cdot S_V \cdot \left(C_{iox} - c_{ox} \right), \tag{S15}$$

where c_{ox} represents the oxygen concentration, D_{ox} denotes the diffusion coefficient for oxygen in the interstitial region, A_{ox} and k_{ox} are parameters associated with oxygen uptake, P_{er} indicates the vascular permeability of oxygen, which governs the diffusion across the walls of tumor vessels, and C_{iox} signifies the oxygen concentration present in the vascular system. The transport of oxygen across the vascular barrier is primarily governed by diffusion, as the contribution of convection is minimal in comparison to diffusion (Popel 1989). In view of the uniform reduction of interstitial fluid pressure observed in tumors, the pressure differentials both within the tissue and across the tumor vessel wall are minimal (Chauhan et al. 2011), leading to an expectation of low Péclet numbers.

Transport of ICBs

Calculation of hydraulic conductivity, vascular permeability and reflection coefficient

According to the theory of particle transport through cylindrical pores, the hydraulic conductivity (L_p) of the vessel walls, vascular permeability (P_{er}) and reflection coefficient (σ_f) can be determined by the size of the pores or the relative size of the particle to the size of the pores, using the following relations (Deen 1987):

$$L_p = \frac{\gamma r_0^2}{8\eta L_{vw}} \tag{S16}$$

$$P_{er} = \frac{\gamma H D_0}{L_{vw}} \tag{S17}$$

$$\sigma_f = 1 - W \tag{S18}$$

in which, γ denotes the proportion of the surface area that is occupied by pores (vessel wall effective porosity), r_0 represents the pore radius, η refers to the viscosity of water at 310 K, and L_{vw} indicates the thickness of the vessel wall. D_0 is recognized as the diffusion coefficient of a particle in a free solution at 310 K, which can be calculated using the Stokes-Einstein equation as:

$$D_0 = \frac{K_b T_{emp}}{6\pi\eta r_s} \tag{S19}$$

where K_b represents the Boltzmann constant, T_{emp} denotes the temperature, and r_s refers to the radius of the diffusing particle. The parameters H and W account for hydrodynamic and steric interactions, respectively. For dilute solutions, they can be calculated by (Deen 1987; Stylianopoulos et al. 2015) :

$$H = \frac{6\pi F}{K_t} \tag{S20}$$

$$W = \frac{F(2-F)K_s}{2K_t} \tag{S21}$$

F is defined as the partition coefficient (Deen 1987):

$$F = 1 - \lambda^2 \tag{S22}$$

where λ denotes the ratio of the drug particle size to the vessel wall pore size, $\lambda = r_s / r_0$. The coefficients K_s and K_t as presented in **Eqs. (11-12)** are determined by (Deen 1987):

$$\begin{pmatrix} K_t \\ K_s \end{pmatrix} = \frac{9}{4}\pi^2\sqrt{2}\left(1-\lambda\right)^{-5/2}\left[1+\sum_{n=1}^{2}\begin{pmatrix} \alpha_n \\ b_n \end{pmatrix}\left(1-\lambda\right)^n\right]+\sum_{n=0}^{4}\begin{pmatrix} \alpha_{n+3} \\ b_{n+3} \end{pmatrix}\lambda^n \tag{S23}$$

For oxygen, both γ and H in **Eq. (8)** were set to unity, assuming that oxygen diffuses freely through the vessel wall without being impeded by hydrodynamic interactions.

Boundary Conditions

Host and tumor tissue boundary conditions

To model spherical growth, we assume symmetry and simulate only one-eighth of the full tumor domain (**Figure S2**). Symmetry is enforced by imposing zero normal displacement (**u**) on each of the three symmetry planes.

$$\mathrm{n} \cdot \boldsymbol{u} = 0 \tag{S24}$$

where n is the outward unit normal.

Consistent with symmetry, zero normal flux boundary conditions are applied on the same planes for oxygen (c_{ox}).

$$-\mathrm{n} \cdot \mathrm{N_i} = 0 \rightarrow -\mathrm{n} \cdot \left(-\mathrm{D_{ox}} \nabla \mathrm{c_{ox}} + \mathbf{v}^{\mathrm{f}} \mathrm{c_{ox}} \right) = 0 \tag{S25}$$

and for interstitial fluid pressure (no normal flow), based on Darcy's law and the solid velocity $\mathbf{v}^s$.

$$-\mathrm{n} \cdot \left(-\mathrm{k_{th}} \nabla \boldsymbol{p}_i + \mathbf{v}^s \right) \tag{S26}$$

Similarly, no flux boundary conditions are imposed on the symmetry planes for immune checkpoint blockers anti-PD-1

$$-\mathrm{n} \cdot \mathrm{N_i} = 0 \rightarrow -\mathrm{n} \cdot \left(-\mathrm{D_{anti\text{-}PD\text{-}1}} \nabla \left[\mathrm{anti\text{-}PD\text{-}1} \right] + \boldsymbol{v}^{\mathrm{f}} \cdot \left[\mathrm{anti\text{-}PD\text{-}1} \right] \right) = 0 \tag{S27}$$

Host tissue boundary conditions

On the external boundary of the host tissue domain, oxygen is prescribed to a constant physiological value representative of normal tissue.

$$\mathrm{c_{ox}} = 0.2\,\mathrm{mol} \cdot \mathrm{m}^3 \tag{S28}$$

A Dirichlet boundary condition is imposed for interstitial fluid pressure on the outer boundaries of the host tissue, so that the pressure takes the value of the fluid pressure in normal tissues.

$$\boldsymbol{p}_{\mathrm{i}} = 0\,\mathrm{Pa} \tag{S29}$$

For drug transport, zero concentration is imposed at the outer boundaries for the anti-PD-1 antibody.

$$c_{0,[\text{anti-PD-1}]} = 0\,\text{mol}\cdot\text{m}^3 \tag{S30}$$

Tumor-host tissue interface conditions

At the tumor-host interface, continuity conditions are imposed. This implies continuity of the cell mass flux across the interface: the normal flux leaving one side must equal the normal flux entering the other side. Accordingly, for the tumor (***t***) and host (***h***) tissue, we impose continuity of the normal mass flux for:

(i) tumor cell populations, including non-stem-like cancer cells (*CCs*). For CCs type ***i***, we apply:

$$(-\mathbf{n})\cdot\left(-D_i\nabla_i + \boldsymbol{v}^s c_i\right)_t = \mathbf{n}\cdot\left(-D_i\nabla_i + \boldsymbol{v}^s c_i\right)_h \tag{S31}$$

(ii) immune cell populations, including natural killer (*NK*) cells, *CD8⁺ T-cells*, *CD4⁺ T-cells*, regulatory T-cells (*Tregs*), and tumor-associated macrophages (*TAMs*) *M1-like TAMs* and *M2-like TAMs*. For each immune cell type ***j***, we apply:

$$(-\mathbf{n})\cdot\left(-D_j\nabla_j + \boldsymbol{v}^s c_j\right)_t = \mathbf{n}\cdot\left(-D_j\nabla_j + \boldsymbol{v}^s c_j\right)_h \tag{S32}$$

Here, **n** denotes the outward unit normal vector on the interface (with opposite directions on the two sides).

Mesh and time step independence assessment

To evaluate the sensitivity of the computations to spatial and temporal discretization, we performed a mesh refinement study and a time-step refinement study. Key outcome measures (i.e. tumor volume and maximum intratumoral anti-PD-1 concentration) were compared at the computational Day 17, one day after the completion of the treatment regimen. The baseline mesh consisted of 6,015 free tetrahedral finite elements and 51,628 degrees of freedom (DOF), whereas the refined mesh consisted of 17,955 elements and 139,361 DOF and the further refined mesh consisted of 33,919 elements and 238,443 DOF. For the time-step analysis, simulations were repeated using identical model settings while reducing the time step size from t = 0.25 d, t = 0.125 d and t=0.0625 d. For each metric M, the relative difference (percent change) between a test setting and the baseline setting was computed as:

$$\%\Delta = 100 \cdot \frac{\left|M_{test} - M_{baseline}\right|}{\left|M_{test}\right|} \tag{S33}$$

where M_{test} denotes the value obtained with the refined or further refined mesh or time step. The results are summarized in **Table S5**. Under mesh refinement, the differences in Day 17 tumor volume relative to the baseline case were 0.25% and 0.26% for the refined and further refined meshes, respectively, while the corresponding differences in the maximum intratumoral ICBs concentration [anti-PD-1] were 0.10% and 0.13%. For the time-step refinement, the corresponding differences were 0.18% and 0.37% for tumor volume and 0.05% and 0.06% for the maximum intratumoral ICBs concentration [anti-PD-1] using time steps of t=0.125 d and t=0.0625 d, respectively. Overall, the small differences observed across the considered spatial and temporal discretizations indicate that the model predictions are independent of both mesh and time-step refinements within the tested ranges.

To further assess the mesh convergence of the pore-radius-dependent response underlying **Figure 6**, tumor volume and PD-1/anti-PD-1 complex concentration were evaluated across the considered vessel wall pore radius (r_o) range using both the baseline and refined meshes. This analysis was performed for the FUS+ICBs treatment at E=30 kPa on post-treatment Day 10, corresponding to the final computational day. The results showed only small differences between the two meshes, with maximum differences of 0.33% for tumor volume and 0.12% for the PD-1/anti-PD-1 complex (**Table S6**). Moreover, the plateau at approximately r_o=100nm and above was consistently reproduced by both meshes (**Figure S3**), supporting the numerical convergence of this feature.

Mesh convergence was additionally examined for the binding-affinity-dependent model response presented in Figure 9 by comparing tumor volume and PD-1/anti-PD-1 complex concentration across the considered anti-PD-1/PD-1 binding affinity ($\mu_{PD\text{-}1/anti\text{-}PD\text{-}1}$) range using the baseline and refined meshes. This analysis was performed for the FUS+ICBs treatment at a fixed anti-PD-1 source term of $\gamma_{ant\text{-}PD\text{-}1}$=1 mol/cm^3/day on post-treatment Day 10, corresponding to the final computational day. The results showed only small differences between the two meshes, with maximum differences of 0.34% for tumor volume and 0.15% for the PD-1/anti-PD-1 complex (**Table S7**). Furthermore, the binding-affinity-dependent trends in both tumor volume and PD-1/anti-PD-1 complex concentration were consistently reproduced by the baseline and refined meshes (**Figure S4**), supporting the numerical convergence of the parameter-dependent response.

Transport regime characterization using the Péclet number

In solid tumors, the interstitial fluid pressure (IFP) is typically elevated and relatively uniform within the tumor interior, resulting in low pressure gradients and consequently, low interstitial fluid velocities (Moradi Kashkooli et al. 2025). Under these conditions, interstitial transport is generally expected to be dominated by diffusion (Koutsi, Mpekris, and Stylianopoulos 2026). To quantify the relative contributions of convection and diffusion to anti-PD-1 transport, the Péclet number was evaluated using the standard advection-diffusion ratio (Rapp 2016):

$$\mathbf{Pe} = \frac{|\mathbf{u}|\,L}{D_{\text{anti-PD-1}}} \tag{S34}$$

where $|\mathbf{u}|$ is the magnitude of the interstitial fluid velocity, L is the characteristic length scale and $D_{\text{anti-PD-1}}$ is the effective diffusion coefficient of the anti-PD-1 antibody in the tumor interstitium. A characteristic length scale of $L=10^{-4}$ m (0.1 mm) was selected to evaluate the relative contributions of convection and diffusion over a small intratumoral distance, rather than across the entire tumor. The same characteristic length scale was used at the tumor center and tumor margin, allowing differences in the calculated Péclet number to reflect spatial variations in the local interstitial fluid velocity.

Using the interstitial velocity field obtained from the flow solution, the Péclet number was evaluated at therapy onset at both the tumor center and tumor margin. At the tumor center**,** $\mathbf{Pe} \approx 1.5\times10^{-3}$, whereas at the tumor margin, $\mathbf{Pe} \approx 1.3\times10^{-2}$**.** The higher Péclet number at the tumor margin reflects the increased local interstitial fluid velocity associated with the larger pressure gradient in this region. Nevertheless, **Pe** remained well below unity at both locations, indicating that diffusion dominates anti-PD-1 transport under the considered conditions, while the relative contribution of convection increases toward the tumor margin.

2 Supplementary Figures and Tables

2.1 Supplementary Figures

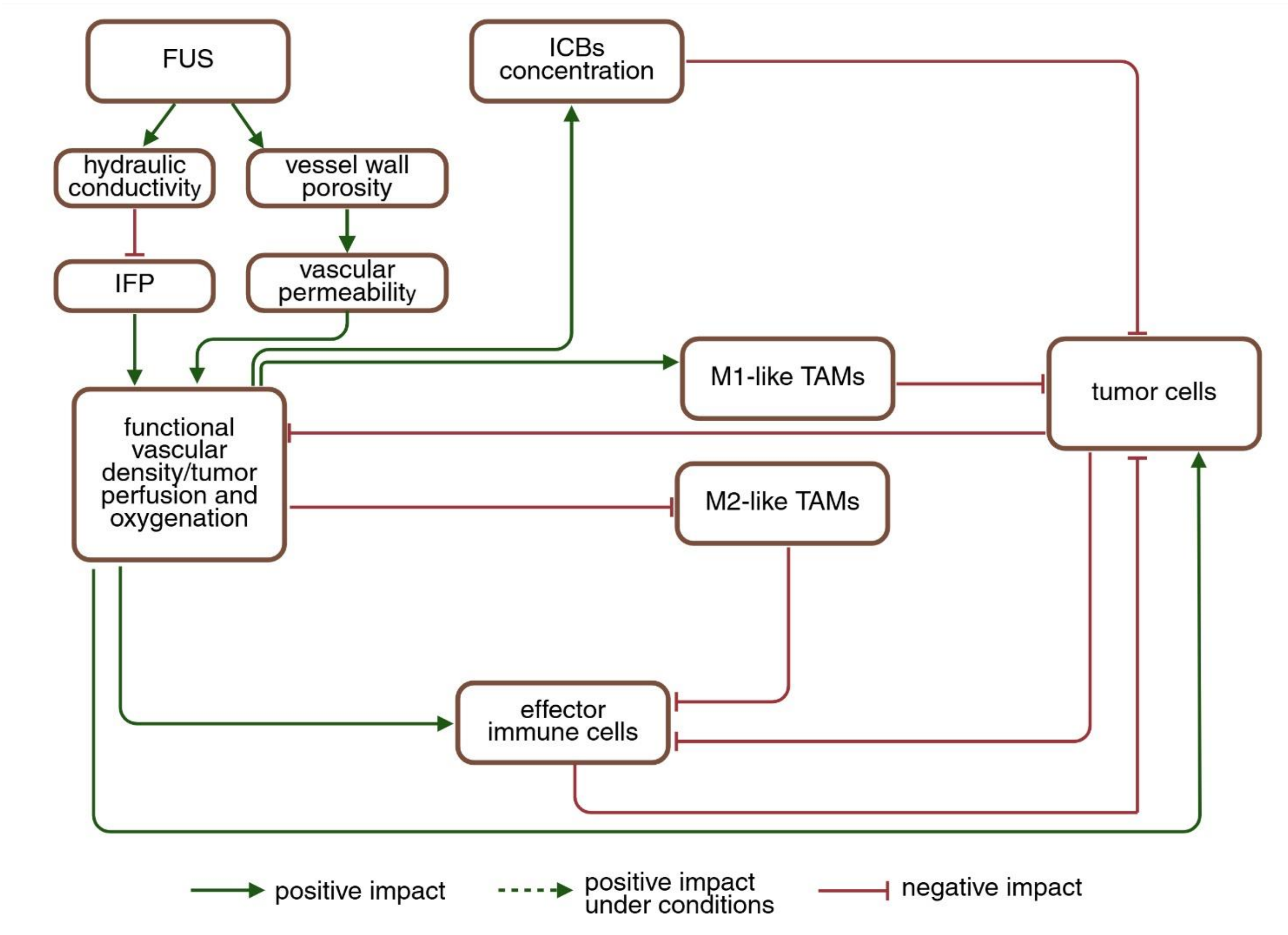

Figure S1. Schematic representation of the mathematical model components and their interconnections. The model includes distinct cell populations each influencing the tumor microenvironment in specific ways. The diagram illustrates how individual components, and their combinations interact to affect functional vascular density, tumor perfusion and oxygenation within the system. These interactions can produce positive, negative, or condition-dependent effects.

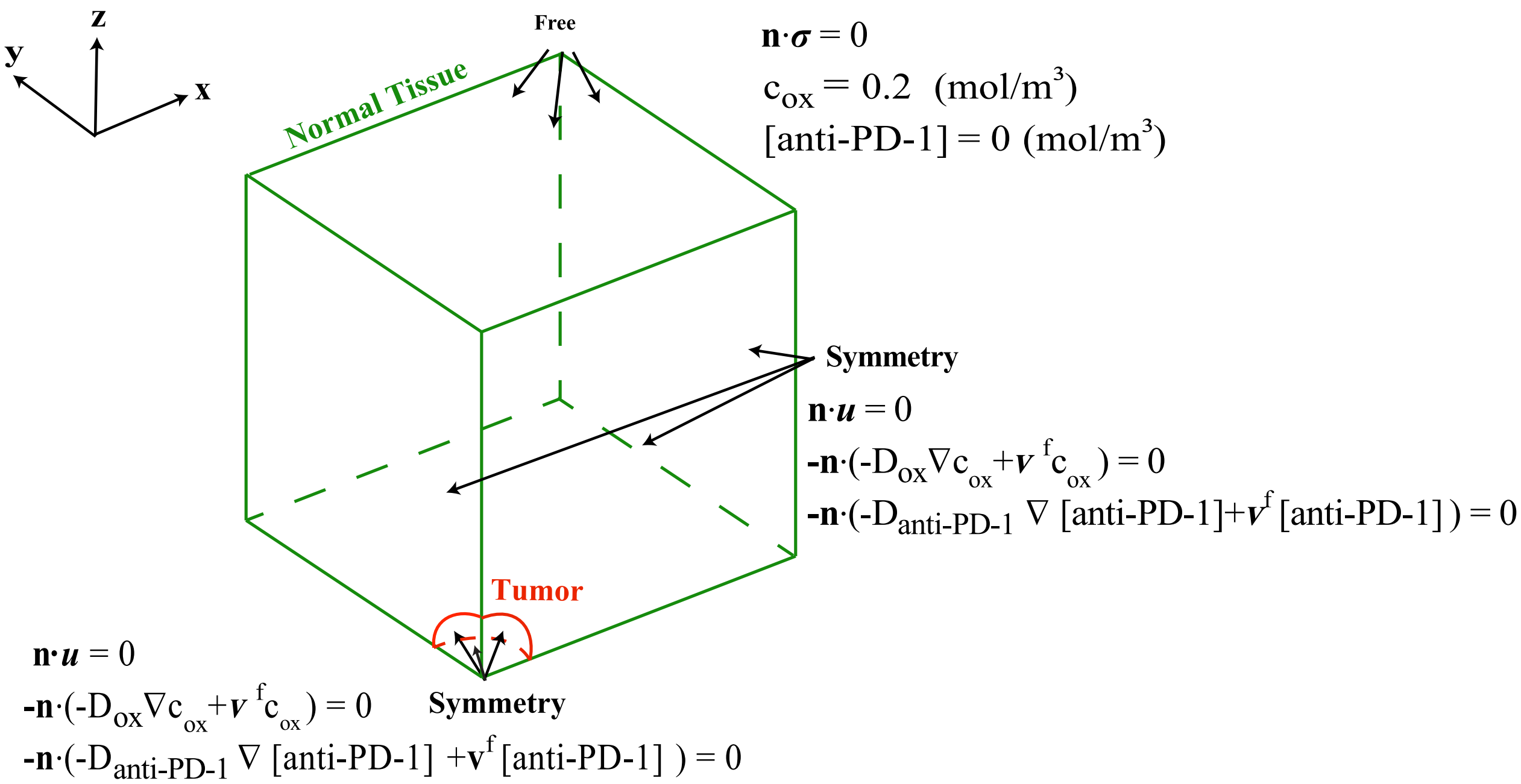

Figure S2. The computational domain along with the specified boundary conditions that have been utilized for the current analysis of stress ($\boldsymbol{\sigma}$), displacement ($\boldsymbol{u}$), the concentration of oxygen (c_{ox}), and the concentrations associated with the anti-PD-1 antibody, specifically denoted as anti-PD-1.

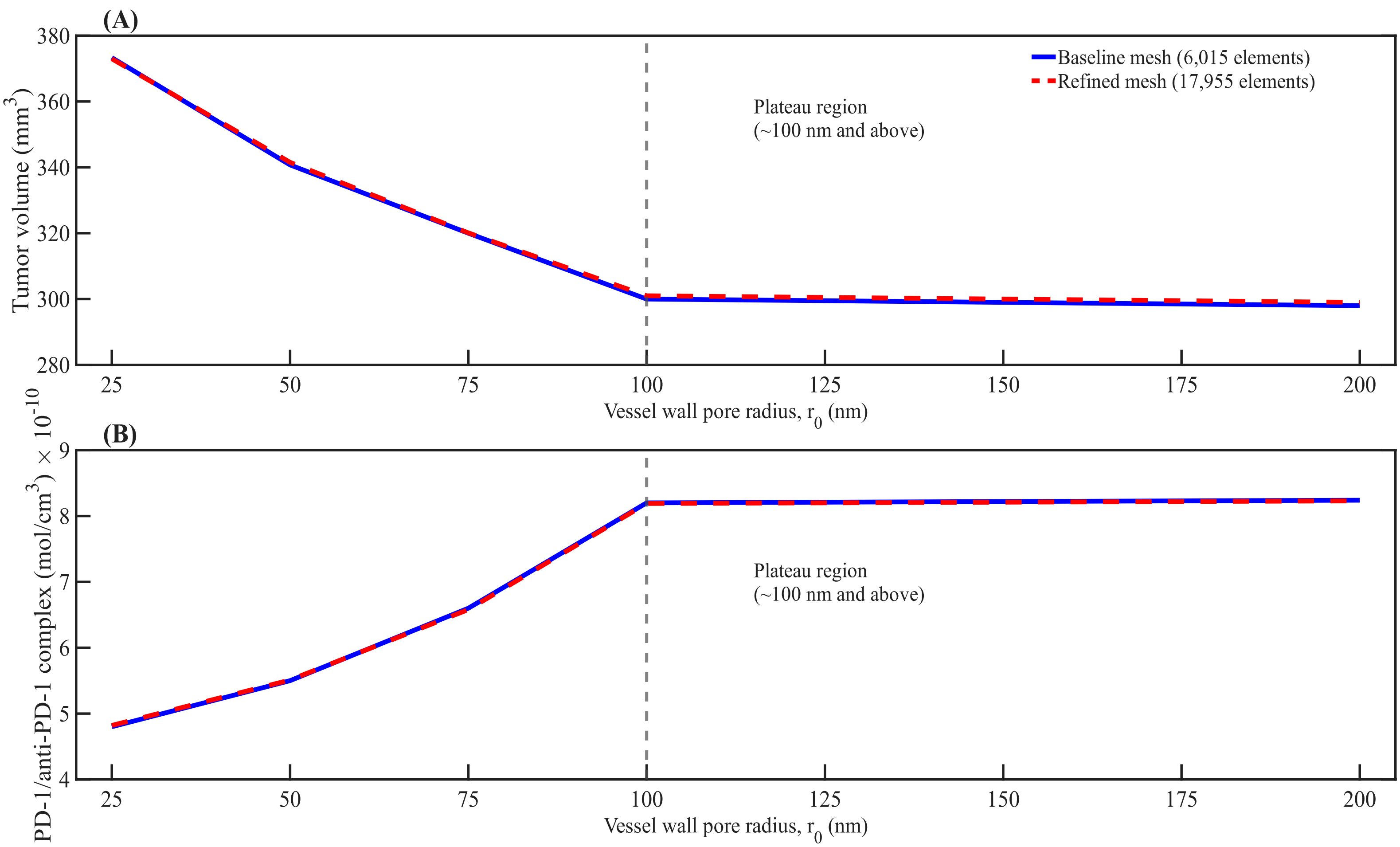

Figure S3. Mesh convergence assessment of the pore-radius-dependent model response. Comparison of results obtained using the baseline mesh (6,015 tetrahedral elements; 51,628 DOF) and the refined mesh (17,955 tetrahedral elements; 139,361 DOF) for the FUS+ICBs treatment at E=60 kPa on post-treatment Day 10, corresponding to the final computational day. **(A)** Tumor volume and **(B)** PD-1/anti-PD-1 complex concentration as a function of vessel wall pore radius (r_o). The refined mesh closely reproduced the baseline results across the considered range, with the plateau at approximately r_o=100 nm and above consistently reproduced by both meshes.

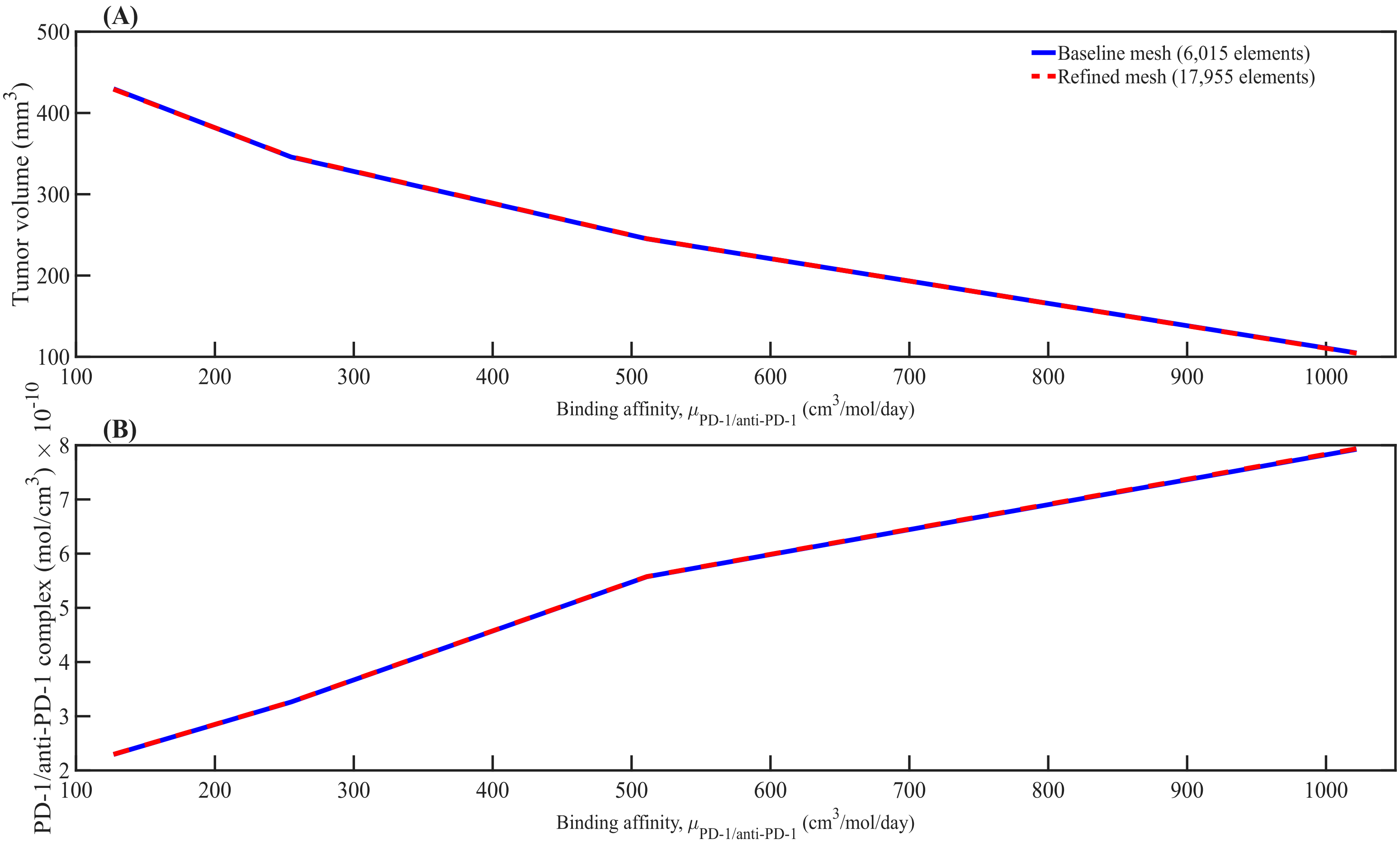

Figure S4. Mesh convergence assessment of the binding-affinity-dependent model response. Comparison of results obtained using the baseline mesh (6,015 tetrahedral elements; 51,628 DOF) and the refined mesh (17,955 tetrahedral elements; 139,361 DOF) for the FUS+ICBs treatment at a fixed anti-PD-1 source term of $\gamma_{anti\text{-}PD\text{-}1}$=1mol/cm³/day on post-treatment Day 10, corresponding to the final computational day. (A) Tumor volume and (B) PD-1/anti-PD-1 complex concentration as a function of the anti-PD-1/PD-1 binding affinity ($\mu_{PD\text{-}1/anti\text{-}PD\text{-}1}$). The refined mesh closely reproduced the baseline results across the considered binding affinity range, confirming the mesh convergence of the parameter-dependent response.

2.2 Supplementary Tables

Table S1. Parameter values used for the incorporation of anti-PD-1 ICBs into the mathematical model, including the interactions among PD-L1, PD-1 and anti-PD-1.

Description	Parameter	Value [Units]	Reference(s)
Amount of PD-L1 on the cancer cells	β_{CCs}	2.18×10^{-12} [mol/g]	(Hadjigeorgiou et al. 2025)
Amount of PD-L1 on the immune cells	β_{IM}	5.22×10^{-7} [mol/g]	(Lai and Friedman 2017; Hadjigeorgiou et al. 2025)
Amount of PD-1 on the immune cells	α_{IM}	7.904×10^{-8} [mol/g]	Hadjigeorgiou, Harkos et al. 2025)
Binding of PD-L1 to the PD-1	$\mu_{\mathrm{PD\text{-}1/PD\text{-}L1}}$	44.5820 [cm^3/mol/day]	(Hadjigeorgiou et al. 2025; Voutouri et al. 2021)
Unbinding of PD-1/PD-L1 complex	$d_{\mathrm{PD\text{-}1/PD\text{-}L1}}$	169.448 [1/day]	(Hadjigeorgiou et al. 2025)
Binding of PD-1 to the anti-PD-1	$\mu_{\mathrm{PD\text{-}1/anti\text{-}PD\text{-}1}}$	511.284 [cm^3/mol/day]	(Hadjigeorgiou et al. 2025)
Unbinding of PD-1/ anti-PD-1 complex	$d_{\mathrm{PD\text{-}1/anti\text{-}PD\text{-}1}}$	0.1 [1/day]	(Voutouri et al. 2021)
Diffusion coefficient of the anti-PD-1 antibody	$D_{\mathrm{anti\text{-}PD\text{-}1}}$	7.87×10^{-6} [m^2/day]	(Siewe and Friedman 2021)

Source term of the anti-PD-1	$\gamma_{\text{anti-PD-1}}$	1 [mol/cm^3/day]	Determined during initial model development
Binding of PD-1 to the anti-PD-1	$\mu_{PD\text{-}1/\text{anti-PD-1}}$	511.284 [cm^3/mol/day]	(Hadjigeorgiou et al. 2025)
Unbinding of PD-1/anti-PD-1 complex	$d_{\text{PD-1/anti-PD-1}}$	0.1 [1/day]	(Voutouri et al. 2021)
Degradation of anti-PD-1	$d_{\text{anti-PD-1}}$	0.1386[1/day]	(Hadjigeorgiou et al. 2025)
Vessel wall thickness	L_{vw}	5×10^{-6} [m]	(Stylianopoulos et al. 2013)
Water viscosity at 310K	η	7×10^{-4} [Pa·s]	(Stylianopoulos et al. 2013)
Absolute temperature	T_{emp}	310.15 [K]	----
Fraction of vessel wall surface area occupied by pores	γ	1×10^{-4} [-]	(Chauhan et al. 2012)
Radius of anti-PD-1 antibody	r_s	6.35 x10^{-9} [m]	(Tykodi et al. 2012)
Half-life of anti-PD- 1 in the body	k_d	2.59 x10^{-2} [1/day]	(Maritaz et al.)
Coefficient for K_t	α_1	-73/60 [-]	(Deen 1987)
Coefficient for K_t	α_2	77.293/50.400 [-]	(Deen 1987)
Coefficient for K_t	α_3	-22.5083 [-]	(Deen 1987)
Coefficient for K_t	α_4	-5.617 [-]	(Deen 1987)
Coefficient for K_t	α_5	-0.3363 [-]	(Deen 1987)

Coefficient for K_t	α_6	-1.216 [-]	(Deen 1987)
Coefficient for K_t	α_7	1.647 [-]	(Deen 1987)
Coefficient for K_s	b_1	7/60 [-]	(Deen 1987)
Coefficient for K_s	b_2	-2.227/50.400 [-]	(Deen 1987)
Coefficient for K_s	b_3	4.0180 [-]	(Deen 1987)
Coefficient for K_s	b_4	-3.9788 [-]	(Deen 1987)
Coefficient for K_s	b_5	-1.9215 [-]	(Deen 1987)
Coefficient for K_s	b_6	4.392 [-]	(Deen 1987)
Coefficient for K_s	b_7	5.006 [-]	(Deen 1987)

Table S2. Parameter values used to describe the components of the tumor microenvironment.

Description	Parameter	Value [Units]	Reference(s)
Cancer cells diffusion coefficient	D_{ell}	$1.5x10^{-11}$ [m^2/s]	(Tracqui 1995; Voutouri et al. 2019)
Killing rate constant of cancer cells by immune cells	K_{rc}	108.913[cm^3/g/day]	(Hadjigeorgiou et al. 2025; Spencer et al. 2021)
Half saturation concentration of PD-1/PD-L1 complex	$K_{PD\text{-}1/PD\text{-}L1}$	8.692 $x10^{-24}$ [mol/cm^3]	(Hadjigeorgiou et al. 2025)
Adaptive immune cells killing potential to innate immune cells	$n_{add2inn}$	4 [-]	(Hadjigeorgiou et al. 2025)
Coefficient for progressive M_1 activation	α_{M1}	0.0002 [1/day^2]	(Mpekris et al. 2020)
Oxygen-dependent activation coefficient of M_1 (controls the sensitivity of M_1 activation to oxygen availability)	k_{M1O2}	0.35 [1/day]	Determined during initial model development
Normalized oxygen activation for M_1 macrophages	$oxrate_{M1}$	$c_{ox}/c_{ox, ref}$ [-]	(Mpekris et al. 2017; Mpekris et al. 2022b)
Reference oxygen concentration	$c_{ox, ref}$	0.2 mol/m^3	(Mpekris et al. 2017; Mpekris et al. 2022b)

Natural degradation/death rate of M_1	m_{M1}	0.02[1/day]	(Mpekris et al. 2020; Mpekris et al. 2022b)
Coefficient for progressive M_2 activation	α_{M2}	5×10^{-5} [1/day^2]	Determined during initial model development
Hypoxia-dependent activation coefficient of M_2 (controls the strength of hypoxia-induced activation)	k_{M2Hyp}	0.45[1/day]	Determined during initial model development
Normalized hypoxia activation for M_2 macrophages	$oxrate_{M2}$	$(1- (c_{ox}/c_{ox,\,ref}))$ [-]	(Mpekris et al. 2017; Mpekris et al. 2022b)
Natural degradation/death rate of M_2	m_{M2}	0.03 [1/day]	Mpekris, Voutouri et al. 2020, Mpekris, Voutouri et al. 2022)
Baseline activation coefficient of NK cells	s_{NKbase}	2.5×10^{-4} [1/d]	(de Pillis, Radunskaya, and Wiseman 2005)
Recruitment coefficient of NK cells	s_{NK}	4.5×10^{-6} [1/d]	Determined during initial model development
Maximum NK population	NK_{max}	2×10^{-4}	(Mpekris, Voutouri et al. 2022
Oxygen-dependent death of NK cells	f_{NK}	$f_{NK0}+(1-(oxrate_{NK})) \times 1.025\times f_{NK0}$<br>range** 0.0412 - 0.0814 [day^{-1}]	(de Pillis, Radunskaya, and Wiseman 2005)
Baseline death rate of NK cells	f_{NK0}	0.0412 [day^{-1}]	(de Pillis, Radunskaya, and Wiseman 2005)

Reduced survival of NK cells under hypoxic conditions	$oxrate_{NK}$	$(1-(c_{ox}/c_{ox,\,ref}))$ [-]	(Mpekris et al. 2017; Mpekris et al. 2022b)
Inhibition coefficient of NK by T_{reg}	$l_{Treg,\,NK}$	100 [$cell^{-1}\cdot day^{-1}$]	(Fouchet and Regoes 2008)
Inhibition coefficient of NK by M_2	$l_{M2,\,NK}$	100 [$cell^{-1}\cdot day^{-1}$]	(Fouchet and Regoes 2008)
Baseline T_{reg} activation coefficient	β_{Treg}	0.0375[1/day]	(de Pillis, Radunskaya, and Wiseman 2005)
Time-dependent recruitment coefficient of T_{reg}	α_{Treg}	0.0005[$1/day^2$]	Determined during initial model development
Natural death coefficient of T_{reg}	m_{Treg}	0.03[1/day]	(Mpekris et al. 2020; Mpekris et al. 2022b)
Therapy-dependent T_{reg} death rate, doubled after treatment initiation.	$(m_{Treg})\cdot(1+if(t>t_{treatment},1,0))$	$(0.03)\cdot(1+if(t>t_{treatment},1,0))$ [1/day]	(Mpekris et al. 2020; Mpekris et al. 2022b)
Source term of C_{d4}	s_{Cd4}	5×10^{-7} [1/day]	Determined during initial model development
Therapy-dependent C_{d4} source term	$(s_{Cd4})\cdot(1+if(t>t_{treatment},1,0))$	$(5\times10^{-7})\cdot(1+if(t>t_{treatment},1,0))$ [1/day]	Determined during initial model development
Maximum population of C_{d4}	C_{d4max}	0.0002 [-]	(Mpekris et al. 2020; Mpekris et al. 2022b)
Natural death rate of C_{d4}	m_{Cd4}	0.001[1/day]	(Fouchet and Regoes 2008)
Source term of C_{d8}	s_{cd8}	2×10^{-5} [1/day]	Determined during initial model development

Maximum population of C_{d8}	C_{d8max}	0.0002 [-]	(Mpekris et al. 2020; Mpekris et al. 2022b)
Oxygen-dependent death of C_{d8} cells	f_{Cd8}	$m_{Cd80}+(oxrate_{Cd8})\times 1.025\times m_{Cd80}$ range **: 0.02 - 0.04 [day^{-1}]	(de Pillis, Radunskaya, and Wiseman 2005)
Baseline death rate of C_{d8}	m_{Cd80}	0.002 [day^{-1}]	(de Pillis, Radunskaya, and Wiseman 2005)
Reduced survival of C_{d8} under hypoxic conditions	$oxrate_{Cd8}$	$(1- (c_{ox}/c_{ox, ref}))$ [-]	(Mpekris et al. 2017; Mpekris et al. 2022b)
Stimulation rate of C_{d8} by C_{d4}	r_{Cd4}	$1x10^{-15}$ [$cells^{-1}.day^{-1}$]	(de Pillis, Radunskaya, and Wiseman 2005)
Stimulation rate of C_{d8} by NK	r_{NK}	1.1×10^{-7} [$cell^{-1}\cdot day^{-1}$]	(de Pillis, Radunskaya, and Wiseman 2005)
Inhibition coefficient of C_{d8} by T_{reg}	$l_{Treg,Cd8}$	100 [$cell^{-1}\cdot day^{-1}$]	(Fouchet and Regoes 2008)
Inhibition coefficient of C_{d8} by M_2	$l_{M2,Cd8}$	100 [$cell^{-1}\cdot day^{-1}$]	(Fouchet and Regoes 2008)
Growth rate parameter	k_1	0.385 [day^{-1}]	Determined during initial model development
Growth rate parameter	k_2	0.0083 [$mol\cdot m^{-3}$]	(Casciari, Sotirchos, and Sutherland 1992)
Hydraulic conductivity	k_{th}	6.5×10^{-10} [$m^2\cdot Pa^{-1}\cdot day^{-1}$] for the host tissue, 6.5×10^{-10} [$m^2\cdot Pa^{-1}\cdot day^{-1}$] for the tumor	(Netti et al. 2000; Papageorgis et al. 2017)

Elastic or Young's modulus	E	21 [kPa] for the host tissue,<br>60 [kPa] for the tumor	Netti, Berk et al. 2000, Samani, Zubovits et al. 2007, Eder, Raith et al. 2014, Mpekris, Panagi et al. 2024, Panagi, Mpekris et al. 2024)
Shear modulus	G, μ	8.75 [kPa] for the host tissue,<br>20.69 [kPa] for the tumor	(Eder et al. 2014; Netti et al. 2000; Samani, Zubovits, and Plewes 2007; Mpekris et al. 2024; Panagi et al. 2024)
Bulk modulus	k	11.67 [kPa] for the host tissue,<br>200 [kPa] for the tumor	(Eder et al. 2014; Netti et al. 2000; Samani, Zubovits, and Plewes 2007; Mpekris et al. 2024; Panagi et al. 2024)
Initial oxygen concentration	C_{iox}	0.2 $[mol \cdot m^{-3}]$	(Mpekris et al. 2017)
Oxygen diffusion coefficient	D_{ox}	1.55×10^{-4} $[m^2 \cdot day^{-1}]$	(Kim, Stolarska, and Othmer 2011)
Oxygen uptake	A_{ox}	2200 $[mol \cdot m^{-3} \cdot day^{-1}]$	(Casciari, Sotirchos, and Sutherland 1992; Kim, Stolarska, and Othmer 2011)
Oxygen uptake	k_{ox}	0.00464 $[mol \cdot m^{-3}]$	(Casciari, Sotirchos, and Sutherland 1992; Kim, Stolarska, and Othmer 2011)

**: linear decrease from maximum to minimum value depending on oxygen levels

Table S3. Initial values of the variables used in the mathematical model at time t=0 day.

Description	Parameter	Initial Value [Units]	Reference(s)
Tumor cell population			
Non-stem-like cancer cells	CCs	0.96 [-]	(Mpekris et al. 2020)
Immune cell populations			
Natural killer cells	NK	0 [-]	(Mpekris et al. 2022a)
$CD8^{+}$ T-cells	C_{d8}	0 [-]	(Hadjigeorgiou and Stylianopoulos 2024)
$CD4^{+}$ T-cells	C_{d4}	0 [-]	(Mpekris et al. 2022a)
Regulatory T-cells	T_{reg}	0.004 [-]	(Mpekris et al. 2022a)
Tumor associated macrophages (TAMs)			
M_1 TAMs	M_1	0.001 [-]	(Mpekris et al. 2022a)
M_2 TAMs	M_2	0.002 [-]	(Mpekris et al. 2022a)
ICBs immunotherapy			
anti-PD-1 concentration	[anti-PD-1]	0 [mol/m^3]	(Hadjigeorgiou et al. 2025)
Other variables			
Oxygen concentration	c_{ox}	0.2 [mol/m^3]	(Mpekris et al. 2017)

Interstitial fluid pressure	$\boldsymbol{p}$	0 [Pa]	(Hadjigeorgiou and Stylianopoulos 2024)
Growth stretch ratio	λ_g	1 [-]	(Hadjigeorgiou and Stylianopoulos 2024)
Solid mechanics, displacement vector	$\boldsymbol{u}$	0 [m]	(Hadjigeorgiou and Stylianopoulos 2024)

Table S4. The value of the parameter k_1, which is employed in the process of fitting the mathematical model to the experimental data for each cancer cell line.

Experimental study	k_1
C6 glioma cells (Shan et al. 2024)	0.33 day^{-1}
4T1 breast cancer cells (Lee et al. 2025)	0.385 day^{-1}

Table S5. Mesh and time-step independence assessment on Day 17, one day after the completion of the treatment regimen. Baseline and refined discretizations are compared using tumor volume and the maximum intratumoral ICBs concentration [anti-PD-1] (mol/cm^3). The mesh-independence assessment considered 6,015, 17,955, and 33,919 tetrahedral elements (51,628, 139,361, and 238,443 DOF, respectively), while the time-step-independence assessment considered time-step sizes of t=0.25, 0.125 and 0.0625 d. Percentage differences relative to the baseline case were calculated using **Eq. (S33)**.

Verification test	Case	Elements	DOF	Time step (days)	Tumor volume (mm^3)	[anti-PD-1] (mol/cm^3)	% change (tumor volume)	% change ([anti-PD-1])
Mesh refinement	Baseline	6,015	51,628	0.25	160.830	$7.931{\cdot}10^{-5}$	--	--
	Refined	17,955	139,361	0.25	161.233	$7.939{\cdot}10^{-5}$	0.25%	0.10%
	Further Refined	33,919	238,443	0.25	161.257	$7.941{\cdot}10^{-5}$	0.26%	0.13%
Time step refinement	Baseline	6,015	51,628	0.25	160.830	$7.931{\cdot}10^{-5}$	--	--
	Refined	6,015	51,628	0.125	161.120	$7.935{\cdot}10^{-5}$	0.18%	0.05%
	Further Refined	6,015	51,628	0.0625	161.428	$7.936{\cdot}10^{-5}$	0.37%	0.06%

Table S6. Mesh-convergence assessment of the pore-radius-dependent model response. Tumor volume and PD-1/anti-PD-1 complex concentration were evaluated at different vessel wall pore radius (r_o) using the baseline mesh (6,015 tetrahedral elements; 51,628 DOF) and the refined mesh (17,955 tetrahedral elements; 139,361 DOF). Simulations correspond to the FUS+ICBs treatment at E=60 kPa and were evaluated on post-treatment day 10, corresponding to the final computational day. Percentage differences between the baseline and refined mesh were calculated according to **Eq. (S33)**. The small differences between the two meshes (≤0.33% for tumor volume and ≤0.12% for the PD-1/anti-PD-1 complex) indicate mesh independent results across the considered r_o range, with the plateau at approximately r_o=100 nm and above consistently reproduced by both meshes.

r_o (nm)	Tumor volume, baseline (mm^3)	Tumor volume, refined (mm^3)	Difference (%)	PD-1/anti-PD-1 complex, baseline (mol/cm^3)	PD-1/anti-PD-1, complex, refined (mol/cm^3)	Difference (%)
25	373.302	372.987	0.08	$4.80 \cdot 10^{-10}$	$4.80336 \cdot 10^{-10}$	0.07
50	340.752	341.520	0.22	$5.50 \cdot 10^{-10}$	$5.50551 \cdot 10^{-10}$	0.10
75	320.000	320.100	0.03	$6.60 \cdot 10^{-10}$	$6.59703 \cdot 10^{-10}$	0.05
100	300.000	301.000	0.33	$8.20 \cdot 10^{-10}$	$8.19 \cdot 10^{-10}$	0.12
200	298.000	299.000	0.33	$8.24 \cdot 10^{-10}$	$8.23 \cdot 10^{-10}$	0.12

Table S7. Mesh-convergence assessment of the binding-affinity-dependent model response. Tumor volume and PD-1/anti-PD-1 complex concentration were evaluated at different anti-PD-1/PD-1 binding affinities ($\mu_{PD\text{-}1/anti\text{-}PD\text{-}1}$) using the baseline mesh (6,015 tetrahedral elements; 51,628 DOF) and the refined mesh (17,955 tetrahedral elements; 139,361 DOF). Simulations correspond to the FUS+ICBs treatment at a fixed anti-PD-1 source term of $\gamma_{ant\text{-}PD\text{-}1}$= 1mol/cm³/day and were evaluated on post-treatment Day 10, corresponding to the final computational day. Percentage differences between the baseline and refined meshes were calculated according to **Eq. (S33)**. The small differences between the two meshes (≤0.34% for tumor volume and ≤0.15% for the PD-1/anti-PD-1 complex) indicate mesh-independent results across the considered binding affinity range, demonstrating that the parameter-dependent response was consistently reproduced by both meshes.

Binding affinity, $\mu_{PD\text{-}1/anti\text{-}PD\text{-}1}$ (cm³/mol/day)	Tumor volume, baseline (mm³)	Tumor volume, refined (mm³)	Difference (%)	PD-1/anti-PD-1 complex, baseline (mol/cm³)	PD-1/anti-PD-1, complex, refined (mol/cm³)	Difference (%)
127	429.720	429.135	0.14	$2.29401 \cdot 10^{-10}$	$2.29653 \cdot 10^{-10}$	0.11
255	345.730	346.100	0.11	$3.26358 \cdot 10^{-10}$	$3.26652 \cdot 10^{-10}$	0.09
511	245.180	245.345	0.07	$5.57362 \cdot 10^{-10}$	$5.57697 \cdot 10^{-10}$	0.06
1022	104.779	104.429	0.34	$7.92496 \cdot 10^{-10}$	$7.93686 \cdot 10^{-10}$	0.15

3 Model Availability

The computational model developed in this study is available on Zenodo: https://doi.org/10.5281/zenodo.21355649.

4. References

Ambrosi, D., and F. Mollica. 2002. 'On the mechanics of a growing tumor', *International Journal of Engineering Science*, 40: 1297-316.

Byrne, H., and L. Preziosi. 2003. 'Modelling solid tumour growth using the theory of mixtures', *Mathematical medicine and biology : a journal of the IMA*, 20: 341-66.

Casciari, J. J., S. V. Sotirchos, and R. M. Sutherland. 1992. 'Variations in tumor cell growth rates and metabolism with oxygen concentration, glucose concentration, and extracellular pH', *J Cell Physiol*, 151: 386-94.

Chauhan, V. P., T. Stylianopoulos, Y. Boucher, and R. K. Jain. 2011. 'Delivery of molecular and nanomedicine to tumors: Transport barriers and strategies', *Annual Reviews Chemical and Biomolecular Engineering*, 2: 281-98.

Chauhan, V. P., T. Stylianopoulos, J. D. Martin, Z. Popovic, O. Chen, W. S. Kamoun, M. G. Bawendi, D. Fukumura, and R. K. Jain. 2012. 'Normalization of tumour blood vessels improves the delivery of nanomedicines in a size-dependent manner', *Nature Nanotechnology*, 7: 383-88.

Ciarletta, P. 2013. 'Buckling instability in growing tumor spheroids', *Phys Rev Lett*, 110: 158102.

de Pillis, L. G., A. E. Radunskaya, and C. L. Wiseman. 2005. 'A validated mathematical model of cell-mediated immune response to tumor growth', *Cancer research*, 65: 7950-58.

Deen, W. M. 1987. 'Hindered Transport of Large molecules in Liquid-Filled Pores', *AIChE J*, 33: 1409-25.

Eder, M., S. Raith, J. Jalali, A. Volf, M. Settles, H. G. Machens, and L. Kovacs. 2014. 'Comparison of different material models to simulate 3-d breast deformations using finite element analysis', *Ann Biomed Eng*, 42: 843-57.

Fouchet, D., and R. Regoes. 2008. 'A population dynamics analysis of the interaction between adaptive regulatory T cells and antigen presenting cells', *PLOS ONE*, 3: e2306.

Hadjigeorgiou, Andreas G., Constantinos Harkos, Aditya K. Mishra, Golnaz Morad, Sarah B. Johnson, Nadim J. Ajami, Jennifer A. Wargo, Lance L. Munn, Triantafyllos Stylianopoulos, and Rakesh K. Jain. 2025. 'Mathematical Modeling and Association Analysis Decipher the Impact of the Gut Microbiome on Cancer Immunotherapy', *Cancer research*, 85: 3139-55.

Hadjigeorgiou, Andreas G., and Triantafyllos Stylianopoulos. 2024. 'Hybrid model of tumor growth, angiogenesis and immune response yields strategies to improve antiangiogenic therapy', *npj Biological Physics and Mechanics*, 1: 4.

Holzapfel, G. A., T. C. Gasser, and R. W. Ogden. 2000. 'A new constitutive framework for arterial wall mechanics and a comparative study of material models', *J. Elasticity*, 61: 1-48.

Kim, Y., M. A. Stolarska, and H. G. Othmer. 2011. 'The role of the microenvironment in tumor growth and invasion', *Progress in biophysics and molecular biology*, 106: 353-79.

Koutsi, Marina, Fotios Mpekris, and Triantafyllos Stylianopoulos. 2026. 'A multiphysics computational model of focused ultrasound-enhanced drug delivery using temperature-sensitive liposomes', *Biomechanics and Modeling in Mechanobiology*, 25: 60.

Lai, Xiulan, and Avner Friedman. 2017. 'Combination therapy of cancer with cancer vaccine and immune checkpoint inhibitors: A mathematical model', *PLOS ONE*, 12: e0178479.

Lee, S. N., J. H. Choi, H. Moon, D. Kim, J. Hong, H. J. Lee, and Y. T. Lim. 2025. 'Focused ultrasound-triggered doxorubicin liposomes reshape tumor microenvironment to boost checkpoint depletion in triple-negative breast cancer model'.

MacLaurin, J., J. Chapman, G. W. Jones, and T. Roose. 2012. 'The buckling of capillaries in solid tumours', *Proc. R. Soc. A*, 468: 4123-45.

Maritaz, C., S. Broutin, N. Chaput, A. Marabelle, and A. Paci. 'Immune checkpoint-targeted antibodies: a room for dose and schedule optimization?'.

Mascheroni, Pietro, Melania Carfagna, Alfio Grillo, DP Boso, and Bernhard A Schrefler. 2018. 'An avascular tumor growth model based on porous media mechanics and evolving natural states', *Mathematics and Mechanics of Solids*, 23: 686-712.

Moradi Kashkooli, Farshad, Ajay Bhandari, Boram Gu, Michael C. Kolios, Mohammad Kohandel, and Wenbo Zhan. 2025. 'Multiphysics modelling enhanced by imaging and artificial intelligence for personalised cancer nanomedicine: Foundations for clinical digital twins', *Journal of Controlled Release*, 386: 114138.

Mow, V. C., S. C. Kuei, W. M. Lai, and C. G. Armstrong. 1980. 'Biphasic creep and stress relaxation of articular cartilage in compression? Theory and experiments', *Journal of Biomechanical Engineering*, 102: 73-84.

Mpekris, F., S. Angeli, A. P. Pirentis, and T. Stylianopoulos. 2015. 'Stress-mediated progression of solid tumors: effect of mechanical stress on tissue oxygenation, cancer cell proliferation, and drug delivery', *Biomech Model Mechanobiol*, 14: 1391-402.

Mpekris, F., J. W. Baish, T. Stylianopoulos, and R. K. Jain. 2017. 'Role of vascular normalization in benefit from metronomic chemotherapy', *Proc Natl Acad Sci U S A*, 114: 1994-99.

Mpekris, F., M. Panagi, A. Charalambous, C. Voutouri, C. Michael, A. Papoui, and T. Stylianopoulos. 2024. 'A synergistic approach for modulating the tumor microenvironment to enhance nano-immunotherapy in sarcomas', *Neoplasia*, 51: 100990.

Mpekris, F., C. Voutouri, J. W. Baish, D. G. Duda, L. L. Munn, T. Stylianopoulos, and R. K. Jain. 2020. 'Combining microenvironment normalization strategies to improve cancer immunotherapy', *Proc Natl Acad Sci U S A*, 117: 3728-37.

Mpekris, F., C. Voutouri, M. Panagi, J. W. Baish, R. K. Jain, and T. Stylianopoulos. 2022a. 'Normalizing tumor microenvironment with nanomedicine and metronomic therapy to improve immunotherapy', *J Control Release*, 345: 190-99.

Mpekris, F., C. Voutouri, P. Papageorgis, and T. Stylianopoulos. 2018. 'Stress alleviation strategy in cancer treatment: Insights from a mathematical model', *Z Angew Math Mech*: 1-12.

Mpekris, Fotios, Chrysovalantis Voutouri, Myrofora Panagi, James W. Baish, Rakesh K. Jain, and Triantafyllos Stylianopoulos. 2022b. 'Normalizing tumor microenvironment with nanomedicine and metronomic therapy to improve immunotherapy', *Journal of Controlled Release*, 345: 190-99.

Netti, P. A., D. A. Berk, M. A. Swartz, A. J. Grodzinsky, and R. K. Jain. 2000. 'Role of extracellular matrix assembly in interstitial transport in solid tumors', *Cancer Res*, 60: 2497-503.

Panagi, M., F. Mpekris, C. Voutouri, A. G. Hadjigeorgiou, C. Symeonidou, E. Porfyriou, C. Michael, A. Stylianou, J. D. Martin, H. Cabral, A. Constantinidou, and T. Stylianopoulos. 2024. 'Stabilizing Tumor-Resident Mast Cells Restores T-Cell Infiltration and Sensitizes Sarcomas to PD-L1 Inhibition', *Clin Cancer Res*, 30: 2582-97.

Papageorgis, P., C. Polydorou, F. Mpekris, C. Voutouri, E. Agathokleous, C. P. Kapnissi-Christodoulou, and T. Stylianopoulos. 2017. 'Tranilast-induced stress alleviation in solid tumors improves the efficacy of chemo- and nanotherapeutics in a size-independent manner', *Sci Rep*, 7: 46140.

Pirentis, Athanassios P, Christiana Polydorou, Panagiotis Papageorgis, Chrysovalantis Voutouri, Fotios Mpekris, and Triantafyllos Stylianopoulos. 2015. 'Remodeling of extracellular matrix due to solid stress accumulation during tumor growth', *Connective tissue research*, 56: 345-54.

Popel, A. S. 1989. 'Theory of oxygen transport to tissue', *Crit Rev Biomed Eng*, 17: 257-321.

Rapp, Bastian. 2016. *Microfluidics: Modeling, mechanics and mathematics*.
Rodriguez, E. K., A. Hoger, and A. D. McCulloch. 1994. 'Stress-dependent finite growth in soft elastic tissues', *J Biomech*, 27: 455-67.
Roose, T., P. A. Netti, L. L. Munn, Y. Boucher, and R. K. Jain. 2003. 'Solid stress generated by spheroid growth estimated using a linear poroelasticity model', *Microvasc Res*, 66: 204-12.
Samani, A., J. Zubovits, and D. Plewes. 2007. 'Elastic moduli of normal and pathological human breast tissues: an inversion-technique-based investigation of 169 samples', *Phys Med Biol*, 52: 1565-76.
Shan, H., G. Zheng, S. Bao, H. Yang, U. D. Shrestha, G. Li, X. Duan, X. Du, T. Ke, and C. Liao. 2024. 'Tumor perfusion enhancement by focus ultrasound-induced blood-brain barrier opening to potentiate anti-PD-1 immunotherapy of glioma', *Translational Oncology*.
Siewe, Nourridine, and Avner Friedman. 2021. 'TGF-β inhibition can overcome cancer primary resistance to PD-1 blockade: A mathematical model', *PLOS ONE*, 16: e0252620.
Skalak, R., S. Zargaryan, R. K. Jain, P. A. Netti, and A. Hoger. 1996. 'Compatibility and the genesis of residual stress by volumetric growth', *J Math Biol*, 34: 889-914.
Spencer, Christine N., Jennifer L. McQuade, Vancheswaran Gopalakrishnan, John A. McCulloch, Marie Vetizou, Alexandria P. Cogdill, Md A. Wadud Khan, Xiaotao Zhang, Michael G. White, Christine B. Peterson, Matthew C. Wong, Golnaz Morad, Theresa Rodgers, Jonathan H. Badger, Beth A. Helmink, Miles C. Andrews, Richard R. Rodrigues, Andrey Morgun, Young S. Kim, Jason Roszik, Kristi L. Hoffman, Jiali Zheng, Yifan Zhou, Yusra B. Medik, Laura M. Kahn, Sarah Johnson, Courtney W. Hudgens, Khalida Wani, Pierre-Olivier Gaudreau, Angela L. Harris, Mohamed A. Jamal, Erez N. Baruch, Eva Perez-Guijarro, Chi-Ping Day, Glenn Merlino, Barbara Pazdrak, Brooke S. Lochmann, Robert A. Szczepaniak-Sloane, Reetakshi Arora, Jaime Anderson, Chrystia M. Zobniw, Eliza Posada, Elizabeth Sirmans, Julie Simon, Lauren E. Haydu, Elizabeth M. Burton, Linghua Wang, Minghao Dang, Karen Clise-Dwyer, Sarah Schneider, Thomas Chapman, Nana-Ama A. S. Anang, Sheila Duncan, Joseph Toker, Jared C. Malke, Isabella C. Glitza, Rodabe N. Amaria, Hussein A. Tawbi, Adi Diab, Michael K. Wong, Sapna P. Patel, Scott E. Woodman, Michael A. Davies, Merrick I. Ross, Jeffrey E. Gershenwald, Jeffrey E. Lee, Patrick Hwu, Vanessa Jensen, Yardena Samuels, Ravid Straussman, Nadim J. Ajami, Kelly C. Nelson, Luigi Nezi, Joseph F. Petrosino, P. Andrew Futreal, Alexander J. Lazar, Jianhua Hu, Robert R. Jenq, Michael T. Tetzlaff, Yan Yan, Wendy S. Garrett, Curtis Huttenhower, Padmanee Sharma, Stephanie S. Watowich, James P. Allison, Lorenzo Cohen, Giorgio Trinchieri, Carrie R. Daniel, and Jennifer A. Wargo. 2021. 'Dietary fiber and probiotics influence the gut microbiome and melanoma immunotherapy response', *Science*, 374: 1632-40.
Stylianopoulos, T., E. A. Economides, J. W. Baish, D. Fukumura, and R. K. Jain. 2015. 'Towards Optimal Design of Cancer Nanomedicines: Multi-stage Nanoparticles for the Treatment of Solid Tumors', *Ann Biomed Eng*, 43: 2291-300.
Stylianopoulos, T., and R. K. Jain. 2013. 'Combining two strategies to improve perfusion and drug delivery in solid tumors', *Proc Natl Acad Sci U S A*, 110: 18632-37.
Stylianopoulos, T., J. D. Martin, M. Snuderl, F. Mpekris, S. R. Jain, and R. K. Jain. 2013. 'Coevolution of solid stress and interstitial fluid pressure in tumors during progression: Implications for vascular collapse', *Cancer research*, 73: 3833-41.
Stylianopoulos, T., A. Yeckel, J. J. Derby, X. J. Luo, M. S. Shephard, E. A. Sander, and V. H. Barocas. 2008. 'Permeability calculations in three-dimensional isotropic and oriented fiber networks', *Phys Fluids (1994)*, 20: 123601.

Taber, L. A. 2008. 'Theoretical study of Beloussov's hyper-restoration hypothesis for mechanical regulation of morphogenesis', *Biomech Model Mechanobiol*, 7: 427-41.

Tracqui, P. 1995. 'From passive diffusion to active cellular migration in mathematical models of tumour invasion', *Acta Biotheor*, 43: 443-64.

Tykodi, Scott S, Julie R Brahmer, Wen-Jen Hwu, Laura Q Chow, Suzanne Louise Topalian, Patrick Hwu, Kunle Odunsi, Luis H Camacho, John S Kauh, and Henry Clement Pitot. 2012. "PD-1/PD-L1 pathway as a target for cancer immunotherapy: Safety and clinical activity of BMS-936559, an anti-PD-L1 antibody, in patients with solid tumors." In.: American Society of Clinical Oncology.

Voutouri, C., N. D. Kirkpatrick, E. Chung, F. Mpekris, J. W. Baish, L. L. Munn, D. Fukumura, T. Stylianopoulos, and R. K. Jain. 2019. 'Experimental and computational analyses reveal dynamics of tumor vessel cooption and optimal treatment strategies', *Proc Natl Acad Sci U S A*, 116: 2662-71.

Voutouri, C., F. Mpekris, P. Papageorgis, A. D. Odysseos, and T. Stylianopoulos. 2014. 'Role of constitutive behavior and tumor-host mechanical interactions in the state of stress and growth of solid tumors', *PLOS ONE*, 9: e104717.

Voutouri, C., and T. Stylianopoulos. 2014. 'Evolution of osmotic pressure in solid tumors', *J Biomech*, 47: 3441-7.

Voutouri, Chrysovalantis, Mohammad Reza Nikmaneshi, C. Corey Hardin, Ankit B. Patel, Ashish Verma, Melin J. Khandekar, Sayon Dutta, Triantafyllos Stylianopoulos, Lance L. Munn, and Rakesh K. Jain. 2021. 'In silico dynamics of COVID-19 phenotypes for optimizing clinical management', *Proceedings of the National Academy of Sciences*, 118: e2021642118.

Xu, G., P. V. Bayly, and L. A. Taber. 2009. 'Residual stress in the adult mouse brain', *Biomech Model Mechanobiol*, 8: 253-62.

Xu, G., P. S. Kemp, J. A. Hwu, A. M. Beagley, P. V. Bayly, and L. A. Taber. 2010. 'Opening angles and material properties of the early embryonic chick brain', *J Biomech Eng*, 132: 011005.
